# Utility of molecular subtypes and genetic alterations for evaluating clinical outcomes in 1029 patients with endometrial cancer

**DOI:** 10.1038/s41416-023-02203-3

**Published:** 2023-02-16

**Authors:** Yuka Asami, Mayumi Kobayashi Kato, Kengo Hiranuma, Maiko Matsuda, Yoko Shimada, Mitsuya Ishikawa, Takafumi Koyama, Masaaki Komatsu, Ryuji Hamamoto, Minoru Nagashima, Yasuhisa Terao, Atsuo Itakura, Takashi Kohno, Akihiko Sekizawa, Koji Matsumoto, Tomoyasu Kato, Kouya Shiraishi, Hiroshi Yoshida

**Affiliations:** 1 Division of Genome Biology, National Cancer Center Research Institute, Tokyo, 104-0045 Japan; 2 Department of Obstetrics and Gynecology, Showa University School of Medicine, Tokyo, 142-8555 Japan; 3 Department of Gynecology, National Cancer Center Hospital, Tokyo, 104-0045 Japan; 4 Department of Obstetrics and Gynecology, Juntendo University Faculty of Medicine, Tokyo, 113-8421 Japan; 5 Department of Experimental Therapeutics, National Cancer Center Hospital, Tokyo, 104-0045 Japan; 6 Division of Medical AI Research and Development, National Cancer Center Research Institute, Tokyo, 104-0045 Japan; 7 Cancer Translational Research Team, RIKEN Center for Advanced Intelligence Project, Tokyo, 103-0027 Japan; 8 Department of Diagnostic Pathology, National Cancer Center Hospital, 104-0045 Tokyo, Japan

**Keywords:** Endometrial cancer, Tumour biomarkers

## Abstract

**Background:**

We investigated the utility of a molecular classifier tool and genetic alterations for predicting prognosis in Japanese patients with endometrial cancer.

**Methods:**

A total of 1029 patients with endometrial cancer from two independent cohorts were classified into four molecular subtype groups. The primary and secondary endpoints were relapse-free survival (RFS) and overall survival (OS), respectively.

**Results:**

Among the 265 patients who underwent initial surgery, classified according to immunohistochemistry, patients with DNA polymerase epsilon exonuclease domain mutation had an excellent prognosis (RFS and OS), patients with no specific molecular profile (NSMP) and mismatch repair protein deficiency had an intermediate prognosis, and those with protein 53 abnormal expression (p53abn) had the worst prognosis (*P* < 0.001). In the NSMP group, mutant *KRAS* and wild-type *ARID1A* were associated with significantly poorer 5-year RFS (41.2%) than other genomic characteristics (*P* < 0.001). The distribution of the subtypes differed significantly between patients with recurrence/progression and classified by sequencing (*n* = 764) and patients who underwent initial surgery (*P* < 0.001). Among patients with recurrence/progression, 51.4% had the opportunity to receive molecular targeted therapy.

**Conclusions:**

A molecular classifier is a useful tool for determining prognosis and eligibility for molecularly targeted therapy in patients with endometrial cancer.

## Background

Endometrial cancer, the fifth most common cancer in women, accounts for an estimated 382,000 new cancer cases and 90,000 deaths annually worldwide [1]. In Japan, during 2018, 17,089 patients were newly diagnosed with endometrial cancer, and 2597 patients with endometrial cancer died [2]. The overall prognosis in patients with endometrial cancer is generally considered favourable, with a 5-year overall survival (OS) of 80%; nevertheless, 15–20% of patients experience recurrence [3]. Outcomes in patients with endometrial cancer and systemic recurrence are poor, with a median survival hardly exceeding 12 months [4]. A risk stratification system based on clinicopathological factors, such as stage, histopathologic type, grade, myometrial invasion, and lymphovascular space invasion, has been used to identify patients with endometrial cancer who are at risk of a poor prognosis [5, 6]. Patients with endometrial cancer are categorised into risk groups to identify those requiring adjuvant treatment [7, 8]; however, risk stratification using clinical factors evaluated after surgery cannot inform decision-making concerning surgical procedures. Given these deficiencies, the development of an accurate diagnosis and risk stratification system for endometrial cancer is required.

In 2013, The Cancer Genome Atlas Endometrial Collaborative Project proposed four different prognostic subtypes based on genomic abnormalities that reflected endometrial cancer tumour biology [9]. Subsequently, a clinically applicable molecular classification system based on immunohistochemistry (IHC) was developed [10–13]. The molecular classification includes four subtypes: (i) DNA polymerase epsilon mutant (*POLE*-mut), (ii) mismatch repair protein deficiency (MMR-D), (iii) protein 53 abnormal expression (p53abn), and (iv) no specific molecular profile (NSMP) [14]. Molecular subtype assignment is highly reproducible and can be performed on diagnostic endometrial biopsy or curettage. Moreover, molecular subtyping is highly concordant with classification based on subsequent hysterectomy specimens [15, 16]. Recently, a molecular classifier has been reported to be useful in distinguishing prognosis in the Caucasian population with endometrial cancer [13, 15, 17]. The 2020 European Society of Gynaecological Oncology, European Society for Radiotherapy and Oncology, and European Society of Pathology guidelines assign risk groups and make treatment decisions according to these molecular subtypes [5].

Endometrial cancer, similar to colorectal cancer and non-small cell lung cancer [18, 19], has a different prognosis depending on ethnicity [20, 21]. Mahdi et al. reported that Asians had a favourable prognosis despite a higher tumour grade and more advanced stage of illness compared to American Indians/Alaska Natives and non-Hispanic White patients with endometrial cancer [22]. Clinicopathological prognostic factors are defined by stage, histology, grade, myometrial invasion, and lymphovascular space invasion, according to the National Comprehensive Cancer Network and European Society for Medical Oncology regardless of ethnicity, although the genetic alterations in endometrial cancer may differ depending on ethnicity. Therefore, molecular classification should consider the biological differences in endometrial cancer according to ethnicity to be broadly applicable to patients with endometrial cancer. However, few studies have described the prognostic value of a molecular classifier in non-Caucasian patients with endometrial cancer.

Furthermore, few studies have focused on the genomic profiles of patients with advanced endometrial cancer. The majority of patients with endometrial cancer are diagnosed at an early stage, and most patients are cured using surgery alone. However, patients with advanced or recurrent endometrial cancer who do not respond to localised therapy, such as surgery or radiotherapy, have a poor prognosis [23, 24]. Treatment options for advanced endometrial cancer have not changed in the last decade. There are limited options for cytoreductive therapy after initial treatment, and no standard options are available for subsequent recurrence and progression. Therefore, evaluating genomic profiles, including the frequency of actionable mutations in advanced endometrial cancer stages, is important to increase options for molecular targeted therapies after relapse to further improve OS in patients with advanced endometrial cancer.

In this study, we investigated the impact of molecular classification on prognosis in >1000 Asian patients with endometrial cancer, including those who underwent initial surgical treatment, and those who recurred or progressed. Furthermore, we aimed to identify novel molecular prognostic factors and indications for molecularly targeted agents by comprehensively decoding genomic changes in patients with endometrial cancer using targeted sequences.

## Materials and methods

### Study design

The study consisted of two independent retrospective cohorts of 1029 Japanese patients with primary endometrial cancer.

The National Cancer Center Hospital (NCCH) cohort included 265 patients, all of whom underwent initial surgery, 99 (37.4%) received adjuvant therapy, and 73 (27.6%) experienced recurrence or progression after the initial surgery.

The Center for Cancer Genomics and Advanced Therapeutic (C-CAT) cohort included 764 patients, all of whom were cases of recurrence or progression; 553 (72.4%) underwent initial surgery, although data were not available for the initial treatment regimen used in 211 patients. In the 705 patients for whom therapeutic records were available, the median number of administered chemotherapy regimens was 2 (range: 1–9 regimens). One patient who overlapped across both cohorts was excluded. The patient characteristics for each cohort are summarised in Table 1.

**Table 1:** Characteristics of Japanese patients with endometrial cancer.

Characteristics	NCCH cohort		C-CAT cohort		*P* value
	(*n* = 265)	(%)	(*n* = 764)	(%)	
*Clinicopathological parameters*					
Age [year] (median, range)	57	(28–89)	63	(25–85)	<0.001*
Histological types					
Endometrioid					
Grade 1	92	(34.7%)	27	(3.5%)	
Grade 2	30	(11.3%)	31	(4.1%)	
Grade 3	76	(28.7%)	43	(5.6%)	
Unknown grade	0	(0.0%)	255	(33.4%)	
Carcinosarcoma	23	(8.7%)	130	(17.0%)	
Serous	18	(6.8%)	132	(17.3%)	
Clear	12	(4.4%)	25	(3.3%)	
Mixed	11	(4.2%)	26	(3.4%)	
Un/de-differentiated	1	(0.4%)	18	(2.4%)	
Neuroendocrine	0	(0.0%)	7	(0.9%)	
Poorly differentiated carcinoma^a^	1	(0.4%)	14	(1.8%)	
Carcinoma	1	(0.4%)	46	(6.0%)	
Others	0	(0.0%)	10	(1.3%)	
Histological grade^b^					<0.001**
Low	122	(46.0%)	58	(7.6%)	
High	143	(54.0%)	405	(53.0%)	
Unclassifiable	0	(0.0%)	301	(39.4%)	
FIGO (2008) stage					–
IA	99	(37.4%)	NA	(–)	
lB	41	(15.5%)	NA	(−)	
ll	22	(8.3%)	NA	(–)	
lllA	16	(6.0%)	NA	(–)	
lllB	4	(1.5%)	NA	(–)	
lllC	58	(21.9%)	NA	(–)	
lVB	25	(9.4%)	NA	(–)	
*Specimens for targeted gene panel*					–
Initial surgery	265	(100.0%)	553	(72.4%)	
Recurrent tumour	0	(0.0%)	209	(27.3%)	
Unknown	0	(0.0%)	2	(0.3%)	
*Type of targeted gene panel*					–
Ion Ampliseq Hotspot and a custom panel	265	(100.0%)	0	(0.0%)	
FoundationOne CDx	0	(0.0%)	749	(98.0%)	
NCC Oncopanel	0	(0.0%)	15	(2.0%)	
*Adjuvant therapy*					–
None	166	(62.6%)	NA	(–)	
Chemotherapy	97	(36.6%)	NA	(–)	
Radiotherapy	2	(0.8%)	NA	(–)	
*Number of chemotherapy lines given (median, range)*	1	(0–3)	2	(1–9)	<0.001*
*Recurrence or progression*					–
None	192	(72.4%)	NA	(–)	
Recurrence	67	(25.3%)	NA	(–)	
Progression	6	(2.3%)	NA	(–)	
*Outcome*					0.833***
Alive	217	(81.9%)	630	(82.5%)	
Death	48	(18.1%)	134	(17.5%)	
Follow-up period [month] (median, range)	61	(3–149)	28	(1–270)	<0.001*

*NCCH* National Cancer Center Hospital, *C-CAT* Center for Cancer Genomics and Advanced Therapeutics, *FIGO* International Federation of Gynecology and Obstetrics, *NA* not available.

*Mann–Whitney’s *U* test; **low grade vs high grade, Chi-squared test; ***Chi-squared test.

^a^The detailed histological subtype or grade according to the 2020 WHO classification was not provided for this case.

^b^Low: endometrioid carcinoma Grade 1 or 2, high: endometrioid carcinoma Grade 3, carcinosarcoma, serous, clear, mixed, and others.

### National Cancer Center Hospital cohort

#### Characteristics

**Fig. 1:**
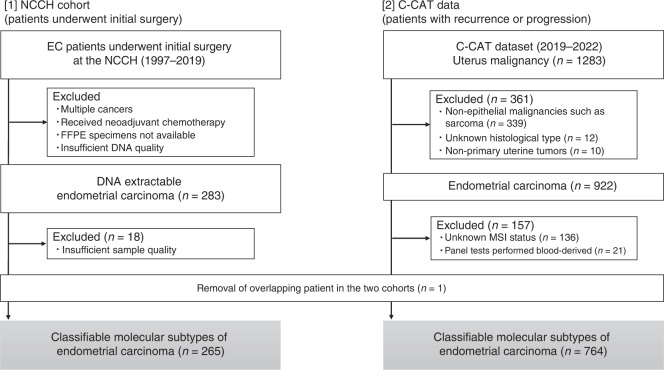
Consort diagram of two cohorts. [1] Two hundred and sixty-five patients with endometrial cancer who underwent initial surgical treatment at our hospital. [2] Seven hundred and sixty-four patients with recurrence or progression from the C-CAT dataset.

Of all the patients who underwent initial surgery at the NCCH between 1997 and 2019 and had a pathological diagnosis of endometrial cancer; 265 were included in the study. Patients who received neoadjuvant chemotherapy were excluded (Fig. 1 [1]). All the cases were reviewed by at least two gynaecological pathologists, and the pathological diagnoses were confirmed according to the 2020 World Health Organization tumour classification. Clinicopathological data, including age and stage (defined by the International Federation of Gynecology and Obstetrics [FIGO] in 2008) were retrospectively obtained for each patient.

#### DNA preparation and next-generation sequencing

Genomic DNA was extracted from 265 hysterectomy formalin-fixed paraffin-embedded (FFPE) endometrial tumour tissue samples using the QIAamp DNA FFPE tissue kit according to the manufacturer’s instructions (Qiagen, Hilden, Germany). DNA obtained from tumour tissues (50 ng) was used for library construction using the Ion AmpliSeq^TM^ Cancer Hotspot Panel v2 and Ion AmpliSeq^TM^ Custom Panel (Thermo Fisher Scientific, Waltham, MA, USA). Experimental details are provided in the Supplementary Methods. Pathological variants in the 50 cancer-related genes were defined using previously reported criteria [25–28] and “high-impact variants,” such as frameshift, stop-gain, stop-loss, and start-loss were defined using SnpEff v4.3 [29] for all exon sequences in the Ion AmpliSeq^TM^ Custom Panel, in addition to pathogenic/oncogenic variants in the ClinVar [30] and OncoKB [31].

#### Classification of molecular subtypes using IHC

Using a molecular classifier, patients with endometrial cancer were divided into four groups: (i) *POLE* exonuclease domain mutation (*POLE*-EDM), (ii) MMR-D, (iii) p53abn and (iv) NSMP. MMR-D and p53abn have been defined in the Supplementary Methods. In this study, all exons of *POLE* were sequenced using the Ion AmpliSeq^TM^ Custom Panel, and we defined *POLE*-EDM as oncogenic/pathogenic, nonsense, and in-frame deletion variants detected within the exonuclease domain of *POLE* that were reported as somatic hotspots [32]. Oncogenic/pathogenic mutations, nonsense mutations, and in-frame deletion mutations that did not contain an exonuclease domain were defined as non-*POLE*-EDM. The molecular classification was conducted as follows: first, tumours were assessed for *POLE*-EDM; next, the presence/deficiency of MMR proteins was assessed using IHC; and finally, tumours were assessed for p53 aberrations using IHC, yielding four subgroups: *POLE*-EDM, MMR-D, p53abn, and NSMP. In this study, double/dual classifier endometrial cancer was classified into upstream branching groups as previously reported [14]. Due to low DNA quality from the FFPE sample, we analysed frozen tumour tissue specimens that were different from the tumour section for MMR-D and p53 IHC in 18 of 265 cases.

### Center for Cancer Genomics and Advanced Therapeutic cohort

#### Characteristics

In June 2019, insurance coverage for the Comprehensive Genome Profiling (CGP) test was introduced in Japan for patients with solid tumours for whom no standard treatment was available or was expected to be completed [33]. The C-CAT was established under the national health insurance system to consolidate, store, and utilise the mutation data and medical information of patients who underwent CGP tests, such as FoundationOne CDx and NCC Oncopanel (https://for-patients.c-cat.ncc.go.jp/). By the end of April 2022, the data of >32,000 patients with advanced cancer had been collected. The definitions of somatic mutations in the C-CAT cohort are provided in the Supplementary Methods.

#### Classification of molecular subtypes using next-generation sequencing

In April 24, 2022, we accessed the C-CAT database (ver. 20220406) and obtained clinical and genomic data for 764 patients with clearly defined endometrial carcinoma, excluding patients with sarcoma (Fig. 1 [2]). C-CAT cases of endometrial cancer were divided into four different molecular subtypes based on genomic abnormalities as follows: (i) *POLE*-EDM, (ii) microsatellite instability high (MSI-H), (iii) *TP53* oncogenic mutation (*TP53*mut), and (iv) NSMP. In endometrial cancer, p53 evaluation using IHC is an excellent surrogate marker for *TP53* mutation status determined by sequencing and has comparable performance and excellent reproducibility among pathologists [34, 35]. Double/dual classification endometrial cancer was classified into upstream branch groups similar to the NCCH cohort [14]. The molecular classification methods by cohort are summarised in the Supplementary Methods.

### Clinical outcomes

In the NCCH cohort, the primary endpoint was relapse-free survival (RFS), which was measured from the date of random assignment to the date of documented relapse or death, whatever the cause. Patients who were alive and relapse-free at the last follow-up were censored. The secondary endpoint was OS, which was measured from the date of random assignment to the date of death, regardless of the cause. Six patients whose tumours could not be completely resected in the initial surgery were excluded from the survival analysis. Patients alive at the time of the analysis were censored at the date of the last follow-up.

In the C-CAT cohort, the primary endpoint was OS. Forty-six patients whose survival period was unknown because the date of diagnosis or last follow-up was not known were excluded from the survival analysis.

### Statistical analysis

Statistical analysis was performed using R software ver. 4.1.2 (R Foundation, Vienna, Austria) and JMP version 16.0.0 software (SAS Institute, New York, USA). Variables that achieved statistical significance in the univariate analysis were subsequently included in the multivariate analysis. The level of statistical significance was set at *P* < 0.05. Cumulative survival was estimated using the Kaplan–Meier method, and differences in survival between two groups was analysed using the log-rank test. The effect of variables on OS or RFS was determined via univariate and multivariate analyses using the Cox proportional hazard model with JMP software.

## Results

### NCCH cohort

#### Characteristics of the patients by molecular subtype

**Fig. 2:**
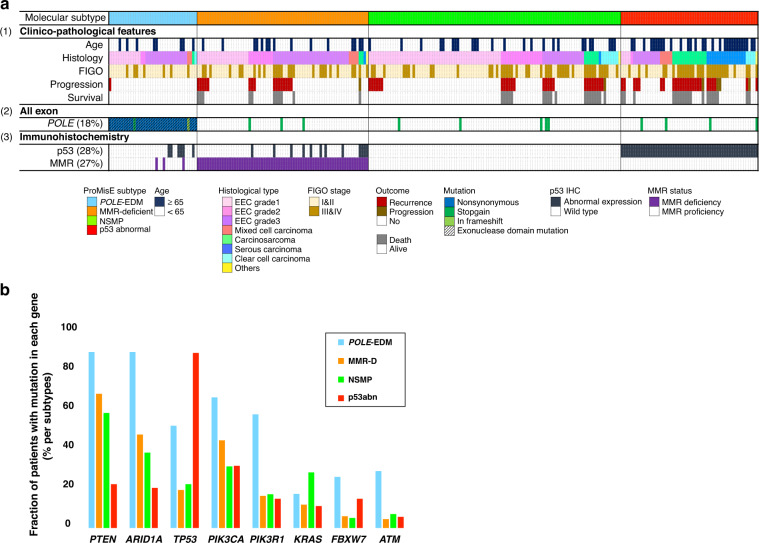
Genetic alteration spectrum by molecular subtype in the NCCH cohort. **a** Clinicopathological factors and molecular subtype in the NCCH cohort. The 265 patients were classified according to (1) histological type and clinicopathological features, (2) somatic mutations used for molecular classification and (3) IHC used for molecular classification. **b** Differences among the four subgroups of recurrently mutant genes. Shown are the mutation frequencies of all genes that were significantly mutated in at least one of the four subgroups.

The clinical characteristics and pathological data of the 265 patients from this cohort are summarised in Table 1. Thirty-six patients were assigned to the *POLE*-EDM group, 70 to the MMR-D group, 103 to the NSMP group, and 56 to the p53abn group (Fig. 2a). The age of the patients in the p53abn group was significantly higher than that of patients in the other molecular subtype groups (*P* < 0.001) (Supplementary Table S1). The distribution of histological types depended on the molecular subtypes, with Grade 3 endometrioid endometrial carcinoma (EEC) prevalent in the *POLE*-EDM and MMR-D groups, low-grade (Grade 1 and 2) EEC in the NSMP group, and serous carcinoma in the p53abn group (*P* < 0.001). The number of progression events was significantly lower in the *POLE*-EDM group and significantly higher in the p53abn group (*P* < 0.001). There was no association between the FIGO stage and molecular subtype. The median follow-up period for all patients was 61 months (range: 3–149 months).

#### Distribution of somatic mutations by molecular subtype

The most frequently mutated gene in patients included in the NCCH cohort was *PTEN*, which was detected in 147/265 (55.5%) patients, followed by *ARID1A*, *TP53*, *PIK3CA* and *PIK3R1*, detected in 112/265 (43.2%), 101/265 (38.1%), 101/265 (38.1%)c and 56/265 (21.1%) patients, respectively. The pattern of somatic mutations varied among different molecular subtypes (Fig. 2b). *PTEN* and *ARID1A* mutations were significantly less frequent in the p53abn group than in the other groups (*P* < 0.001). *KRAS* mutations were found more frequently in patients with endometrial carcinoma and NSMP than in those with other subtypes. There were no typical mesonephric-like adenocarcinomas in 48 patients with *KRAS* mutations. *TP53* mutations were significantly more common in the p53abn group than in the other three groups. This distribution of somatic mutations by molecular subtype was similar to that of the histological type. *TP53*mut/p53 wild-type (wt) was more common in endometrioid carcinoma, while *TP53*wt/p53abn was more common in non-endometrioid carcinoma, and the histological types differed significantly between the two groups (*P* < 0.001) (Supplementary Table S2).

#### Correlation between molecular subtype and clinical outcomes

**Fig. 3:**
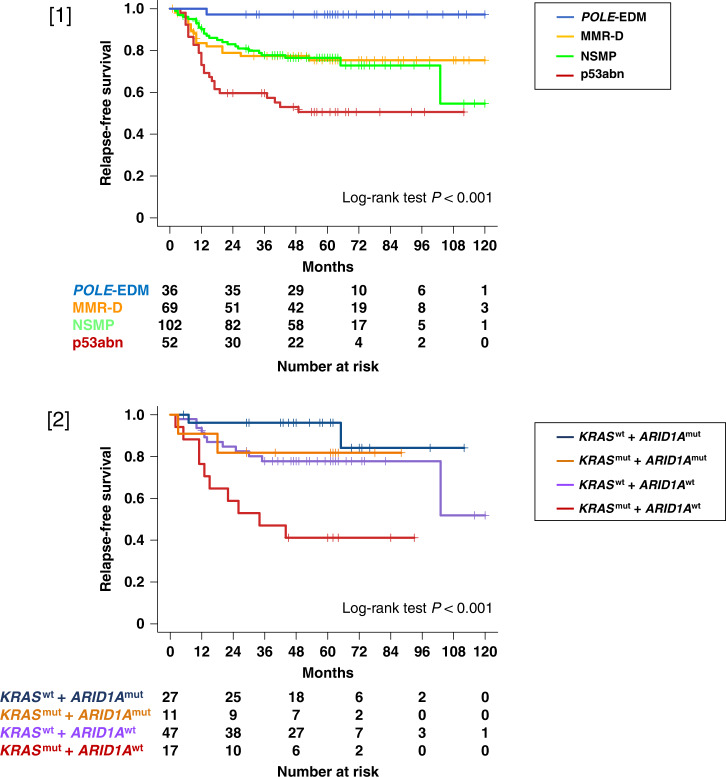
Kaplan–Meier survival curves in the NCCH cohort. [1] RFS according to molecular subtype in all stages of *POLE*-EDM (blue line), MMR-D (orange line), NSMP (green line) and p53abn (red line). [2] RFS according to *KRAS* and *ARID1A* status in patients with endometrial cancer with no specific molecular profile (NSMP). *KRAS* wt and *ARID1A* mut (navy blue line), *KRAS* mut and *ARID1A* mut (dark orange line), *KRAS* wt and *ARID1A* wt (purple line) and *KRAS* mut and *ARID1A* wt (dark red line). mut mutant, wt wild type.

The survival probability for the entire NCCH cohort of endometrial cancer patients is shown in Supplementary Fig. S1. Patients with endometrial cancer with *POLE*-EDM had the best prognosis in terms of RFS and OS; those with MMR-D and NSMP exhibited intermediate prognosis, with no significant difference between the two groups; and those with p53abn had the worst prognosis (Fig. 3 [1]) and Supplementary Fig. S2). In the multivariate analysis performed using a Cox proportional hazards model, patients with endometrial cancer and p53abn were independently found to have a worse RFS than those with *POLE*-EDM (hazard ratio [HR] = 16.2, 95% confidence interval [CI] = 2.16–121.0, *P* = 0.007) (Supplementary Table S3). Patients with endometrial cancer with *POLE*-EDM had a favourable prognosis, with a 100% 5-year RFS rate and 5-year OS rate despite the advanced FIGO Stage (III–IV) (Supplementary Fig. S3 [1], [2]). In the early stage, patients with *POLE-*EDM endometrial cancer had a favourable prognosis, those with MMR-D and NSMP had an intermediate prognosis, and those with p53abn had an unfavourable prognosis, although the differences were not significant (Supplementary Fig. S3 [3], [4]). In the multivariate analysis performed using a Cox proportional hazards model, patients with endometrial cancer with mutant *PTEN* and *ARID1A* had a better RFS than those with wild-type *PTEN* and *ARID1A* (HR = 0.42, 95% CI = 0.25–0.69, *P* < 0.001; HR = 0.58, 95% CI = 0.35–0.97, *P* = 0.040, respectively) (Supplementary Table S3). In 198 patients with EEC, those with *PTEN* mutations had better RFS than those without mutations, with no significant difference in OS (Supplementary Fig. S4).

Forty-eight of the 265 patients had *POLE* mutations, 36 with mutations in the exonuclease domain (P286R/C, 18 patients; V411L, 7 patients; A456P, 5 patients; S297F/Y, 2 patients; S459F/del, 2 patients; F367C, 1 patient; W369*, 1 patient) and 12 with mutations in the non-exonuclease domain (Supplementary Fig. S5A). Patients with exonuclease domain mutations in *POLE* had significantly better RFS and OS than those with non-exonuclease domain mutations in *POLE* (*P* < 0.001) (Supplementary Fig. S5B).

#### Somatic mutations as prognostic factors in patients with no significant molecular profile

Among the 103 patients with NSMP, 17 with *KRAS* mutation and without *ARID1A* mutation had unfavourable RFS (log-rank test; *P* < 0.001) and OS (log-rank test; *P* = 0.002) (Fig. 3 [2] and Supplementary Fig. S6). In the Cox proportional hazard model analysis, patients with mutant *KRAS* and wt *ARID1A* had the worst RFS when wt *KRAS* and mutant *ARID1A* were used as the reference (HR = 6.98, 95% CI = 1.47–33.2, *P* = 0.015) (Supplementary Table S4). The clinical characteristics of the 17 patients with *KRAS* mutation and without *ARID1A* mutation are summarised in Supplementary Table S5.

### C-CAT cohort

#### Patient characteristics

The clinical characteristics and pathological data of the 764 patients with recurrence or progression of endometrial cancer are summarised in Table 1. Sixteen patients were assigned to the *POLE*-EDM group, 87 to the MSI-H group, 267 to the NSMP group, and 394 to the *TP53*mut group (Supplementary Table S6). The C-CAT data showed a high frequency of low-grade endometrial cancer in the NSMP group and high-grade endometrial cancer in the *TP53*mut group, although the histological grades were not available for many patients (Supplementary Table S6). The genetic alterations by molecular subtype for the C-CAT cohort are shown in Supplementary Fig. S7A. In the C-CAT cohort, similar to the NCCH cohort, the distribution of many genetic alterations differed among the four subtypes (Supplementary Fig. S7B). *PTEN*, *ARID1A* and *PIK3CA* mutations were more common in the *POLE*-EDM and MSI-H groups, *KRAS* mutations were more common in the NSMP group, and *PPP2R1A* mutations were more common in the *TP53*mut group (*P* < 0.001). There was no significant association between molecular subtype and histological grade in the 463 patients for whom histological grades were available in the C-CAT cohort (*P* = 0.394).

#### Correlation between molecular subtype and clinical outcome and potential therapeutic targets

**Fig. 4:**
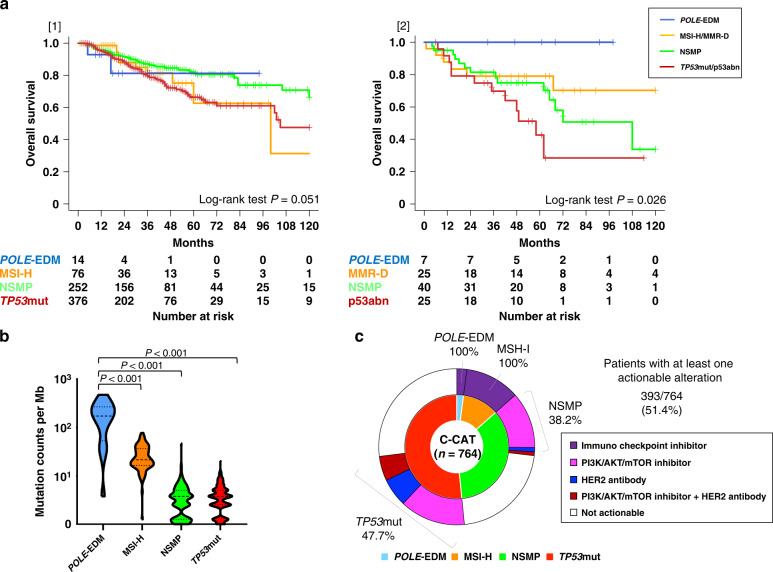
Patient outcomes and therapeutic targets in the C-CAT cohort. **a** Kaplan–Meier survival curves. [1] OS according to molecular subtype in the C-CAT cohort of *POLE*-EDM (blue line), MSI-H (orange line), NSMP (green line), and *TP53*mut (red line). [2] OS according to molecular subtype in advanced Stages (III–IV) among the NCCH cohort of *POLE*-EDM (blue line), MMR-D (orange line), NSMP (green line), and p53abn (red line). **b** Distribution of the tumour mutation burden by four subtypes in the C-CAT cohort. **c** Frequency of actionable mutations by subtypes in the C-CAT cohort.

The survival probabilities for the entire C-CAT cohort of endometrial cancer patients are shown in Supplementary Fig. S1. In patients with endometrial cancer who had recurrence or progression, OS showed no significant difference among the four subtypes (Fig. 4a [1]). Among patients with advanced disease (FIGO Stage III–IV) who underwent initial surgery, OS was classified into four subtypes (Fig. 4a [2]). The tumour mutation burden (TMB), obtained from C-CAT data, was significantly higher in the *POLE*-EDM group than in the other three groups (Fig. 4b). Among the 764 patients in the C-CAT cohort, there were 393 patients (51.4%) with at least one actionable alteration (Fig. 4c).

### Comparison of molecular subtypes and mutation patterns between patients who underwent initial surgery and patients with recurrence or progression

**Fig. 5:**
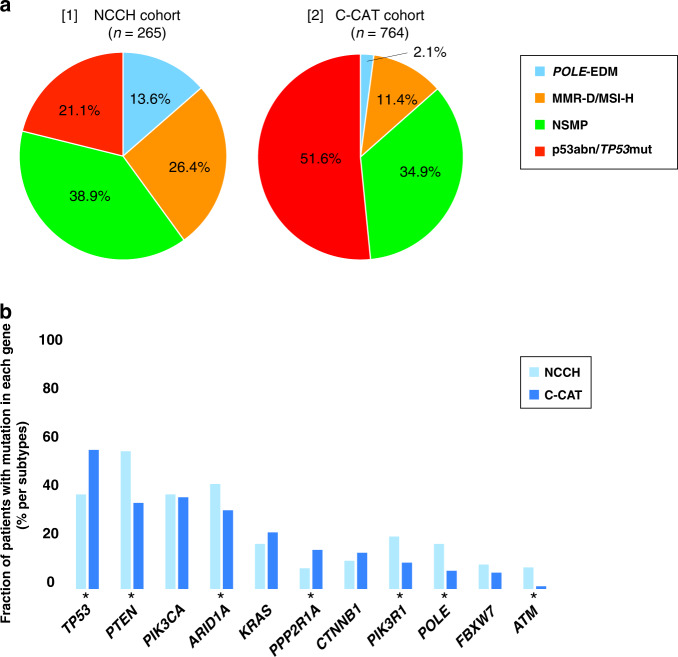
Differences in molecular subtypes and gene frequencies between the NCCH and C-CAT cohorts. **a** Frequency of molecular subtypes in the NCCH cohort (initial surgery patients) vs. the C-CAT (recurrence or progression patients). **b** Differences between the two cohorts of recurrent mutant genes. Asterisks indicate genes with significant differences between the two groups.

In patients with recurrence or progression, the frequency of occurrence of *POLE*-EDM in the C-CAT cohort was significantly lower than that in the NCCH cohort, in contrast to the frequency of *TP53*mut, which was significantly higher (2.1% vs. 13.6% and 51.2% vs. 21.1%, respectively; *P* < 0.001; Fig. 5a). The patients with recurrence or progression had a significantly lower frequency of *PTEN* and *ARID1A* mutations and higher frequency of *TP53*mut than patients who underwent initial surgery (Fig. 5b). Conversely, the frequency of *PIK3CA*, *KRAS* and *CTNNB1* mutations was not different between the two cohorts.

The genetic alterations by cohort in Japanese patients with *POLE-*EDM endometrial cancer are shown in Supplementary Fig. S8A. The frequency of gene mutations did not differ between the two cohorts (Supplementary Fig. S8B). Fifty-six of the 764 patients from the C-CAT cohort had *POLE* mutations, including 16 with mutations in the exonuclease domain (P286R, 6 patients; V411L, 6 patients; S297F, 1 patient; P436R, 1 patient; A456P, 1 patient; S459F, 1 patient), and 40 with mutations in the non-exonuclease domain (Supplementary Fig. S9A). In the C-CAT cohort, there was no significant difference in OS between patients with exonuclease mutations in *POLE* and those with non-exonuclease mutations in *POLE* (*P* = 0.733) (Supplementary Fig. S9B). OS rates according to molecular subtype for all patients by each cohort are shown in Supplementary Fig. S10. The analysis of the correlation between molecular subtype and outcome in both cohorts, excluding histologic types for which the molecular classification algorithm is not valid (carcinosarcoma, undifferentiated/differentiated carcinoma, neuroendocrine carcinoma, and carcinomas of unknown histology), showed that endometrial carcinoma with abnormal p53 expression/*TP53* mutation exhibited poor prognostic behaviour (Supplementary Fig. S11).

## Discussion

We demonstrated that molecular classification can be useful for determining prognosis in patients with endometrial cancer who underwent initial surgery, regardless of ethnicity. As previously reported, endometrial cancer with p53abn was associated with an unfavourable prognosis, and since the p53abn was a poor prognostic factor independent of FIGO stage and histological grade, the molecular classifier system included prognostic factors independent of the stages and histological types. Patients with NSMP (as identified by an IHC-based on molecular classifier) endometrial cancer with mutant *KRAS* and wt *ARID1A* showed poor RFS compared with those with other *KRAS*/*ARID1A* statuses. Conversely, there were significantly fewer patients classified as *POLE*-EDM and significantly more patients classified as *TP53*mut among patients with recurrence or progression than among those who underwent initial surgery, and the frequency of molecular subtypes was clearly different between the two groups. Furthermore, in patients with endometrial cancer with recurrence or progression, the molecular subclassification based on comprehensive genomic analysis in the C-CAT database could enable appropriate treatment of recurrence, and ~50% of patients may be eligible for molecularly targeted therapies such as immune checkpoint inhibitors.

Somatic alterations in the exonuclease domain of *POLE* occur in a subgroup of endometrial cancers with ultra-mutation (frequently ≥100 mutations/Mb) [9] and excellent clinical outcome [9, 11–13]; the former was replicated in the C-CAT cohort and the latter in the NCCH cohort. The high mutation burden results in an enriched antigenic neoepitope and enhanced anti-tumour immune response, suggesting a favourable prognosis for this subtype [36, 37]. We first reported that the frequency of *POLE*-EDM was significantly lower in the recurrence or progression group than in the initial surgery group, suggesting that there may not be a good prognosis after recurrence. Since only 2.1% of patients with endometrial cancer with recurrence or progression presented with *POLE*-EDM tumour characteristic behaviour, these findings support the omission of adjuvant treatment and a decrease in surveillance for Asian patients with *POLE*-EDM endometrial cancer. In addition, patients with *POLE*-EDM endometrial cancer have significantly higher TMB than those with other subtypes; therefore, appropriate treatment such as immune checkpoint inhibitor therapy may improve prognosis [38].

Patients with MMR-D endometrial cancer had an intermediate prognosis in the NCCH cohort. Both *POLE*, which controls base incorporation and proofreading, and the MMR system, which monitors post-replication, play central roles in facilitating accurate DNA replication [39]; therefore, somatic mutations in *POLE-* or MMR-related genes (*MLH1*, *MSH2*, *MSH6* and *PMS2*) result in DNA replication repair deficiency. However, endometrial cancers with MMR-D were reported to have lower TMB and neoantigen loads than those with *POLE* mutations [36], which might explain the difference in prognosis between the two groups. Pembrolizumab, an anti-PD-1 monoclonal antibody, demonstrated robust and durable anti-tumour activity with manageable toxicity and promising survival in patients with advanced MSI-H/MMR-D endometrial cancer [40].

Among the four groups in the NCCH cohort, the highest number of patients were classified in the NSMP group, with no molecular characteristics for molecular classification. In the NCCH cohort, we identified mutant *KRAS* and wt *ARID1A* as novel biomarkers associated with poor prognosis in the NSMP group. Mutant *KRAS* and wt *ARID1A* may identify high-grade or advanced-stage disease characterised by aggressive behaviour in the NSMP population. *KRAS* mutations are found in 10–30% of well-differentiated endometrial cancers and are known for their role as an early checkpoint in the transition from hyperplasia to cancer [41, 42] and as markers of invasive potential in well-differentiated tumours [42, 43]. However, there is no consensus on how *KRAS* mutation affects the prognosis of endometrial cancers, with only a few reports associating it with poor prognosis [44] and aggressive clinical behaviour [45]. *ARID1A* mutations are frequently detected in patients with endometrial cancer, and cell line assays have indicated increased tumorigenicity when *ARID1A* mutations are combined with other genomic abnormalities [46, 47]. However, a study that used The Cancer Genome Atlas dataset reported that *ARID1A* mutation alone was associated with better prognosis in patients with endometrial cancer compared to wt *ARIDIA* [47]. These mechanisms need to be elucidated in the future. A total of 38.2% of patients with recurrence or progression classified as NSMP had *PIK3CA* oncogenic mutations or *ERBB2* alterations, suggesting that they may be candidates for molecular targeted therapies such as PI3K/AKT/mTOR inhibitors and HER2 antibody drugs.

Patients with p53abn endometrial cancer had significantly worse clinical outcomes than those with other molecular subtypes in the NCCH cohort. However, patients with endometrial cancer with p53abn with *POLE*-EDM or MMR-D have been reported to have a favourable prognosis [14]. *TP53*mut is associated with more aggressive tumours and poor overall outcome in various cancer types compared to wt *TP53* [48], including endometrial cancers, and ~27.8% of patients with endometrial cancer have been reported to have *TP53*mut regardless of histological type [49]. In a comparison between the C-CAT and NCCH cohorts, the percentage of *TP53* abnormalities was higher in patients with metastatic progression or recurrence (51.6%) than in those who underwent initial surgery (21.1%). Patients with *TP53*mut exhibited a trend toward a worse prognosis than those with other subtypes in the recurrence or progression patient cohort. This suggests that patients with *TP53* abnormalities should be carefully surveilled, considering their susceptibility to recurrence.

In the C-CAT cohort, approximately half of the patients with *TP53*mut (47.7%) had at least one genetic alteration that could be a potential therapeutic target. Although there have been few studies on molecular targeted therapy for patients carrying *TP53*mut, a therapy for targeting a neoantigen derived from a common *TP53*mut has been reported [50]. Recently, Leslie et al. found that bevacizumab plus chemotherapy more significantly improved OS in patients with advanced endometrial cancer with *TP53*mut compared with those without *TP53*mut [51]. These results indicate that precision medicine for uterine cancer is expected to advance in the future.

The sensitivity, specificity, and diagnostic odds ratio for detecting abnormal *TP53* by IHC in this study were 0.60 (95% CI 0.54–0.65), 0.92 (95% CI 0.87–0.95) and 16.2 (95% CI 8.05–32.5), respectively. Compared to the results of previous studies [34], the sensitivity of p53 IHC appears to be low, and a combination of factors may explain this finding. We analysed frozen tumour tissue specimens that were different from the tumour section used for p53 IHC in 18 of 265 cases because of low-quality DNA from the FFPE sample. In these cases, the tumour section used for IHC was different from the tumour tissue used for *TP53* sequencing; therefore, intratumoral heterogeneity of *TP53* mutations may result in a discrepancy between p53 status determined by IHC and the *TP53* sequencing results. The small number of subclones with *TP53* mutations, the small number of cases with *TP53* mutations showing wild-type staining patterns in IHC, and technical issues with IHC may also explain this discrepancy.

We consider that the inability of the molecular classification of the C-CAT cohort to predict prognosis is explained by the fact that patients with Stage I–II endometrioid endometrial carcinoma do not qualify for sequencing by the C-CAT programme because of lack of recurrence or progression of the disease. The prognostic impact of molecular classification of recurrent/advanced endometrial cancer requires further investigation. Moreover, it is important to note that *POLE*-EDM tumour is not a favourable prognostic factor in advanced stage and metastatic disease. In aggressive tumours, we speculate that as the tumour progresses, the clones survive by acquiring favourable features for survival, such as tumour immune escape mechanisms; however, further studies are required to determine the reason for this finding.

In conclusion, endometrial cancer molecular subtypes represent a useful classification system that evaluates tumour characteristics regardless of ethnicity, with the potential to predict the prognosis of patients who underwent initial surgery and determine the usefulness of appropriate molecular targeted therapy for patients with recurrence or progression. Furthermore, patients with NSMP endometrial cancer and combined mutant *KRAS* and wt *ARID1A* who underwent initial surgery have a poor prognosis. These results indicate that molecular classification can distinguish patients with similar histological features but different prognoses, as well as guide therapeutic strategies and appropriate surveillance.

## Supplementary information

Supplementary_file

REMARK

## Data Availability

The data generated in this study are available upon reasonable request from the corresponding authors.
