# Supplementary material for: Utility of molecular subtypes and genetic alterations for evaluating clinical outcomes in 1029 patients with endometrial cancer
Source: Br J Cancer. 2023 Feb 16;128(8):1582–91. doi: 10.1038/s41416-023-02203-3 (PMC10070437; doi:10.1038/s41416-023-02203-3)
Supplement: Supplementary file 1 — Supplementary_file [file 41416_2023_2203_MOESM1_ESM.pdf]

Utility of molecular subtypes and genetic alterations for evaluating clinical outcomes in 1,029 patients with endometrial cancer

Asami Y et al.

Supplementary Methods

Quality control for next-generation sequencing in the NCCH cohort

Genomic DNA was extracted from 265 formalin-fixed paraffin-embedded (FFPE) tumor tissues using a QIAamp DNA FFPE tissue kit according to the manufacturer's instructions (Qiagen, Hilden, Germany). Eighteen of the 265 samples consisted of low-quality DNA extracted from FFPE-derived tumor tissues, thus DNA was extracted from snap-frozen tumor tissues using a QIAamp DNA Mini Kit. Purified genomic DNA obtained from tumor tissues (50 ng) was used for library construction using the Ion AmpliSeq™ Cancer Hotspot Panel v2 (Thermo Fisher Scientific, Waltham, MA, USA), which targets approximately 2,800 COSMIC mutational hotspot regions of 50 cancer-related genes. Sequencing was performed using the Ion Proton platform (Thermo Fisher Scientific). An Ion AmpliSeq™ Custom Panel, which was designed for *PTEN* (coverage rate: 89.2%), *ARID1A* (coverage rate: 97.3%), *TP53* (coverage rate: 100%), *POLE* (coverage rate: 100%), *PIK3R1* (coverage rate: 99.6%), and *PPP2R1A* genes (coverage rate: 100%) using Ion AmpliSeq™ Designer (https://www.ampliseq.com), was also used (Solution ID: IAD191594_167). For sample quality control, samples with a mean read depth coverage >1000 and a base quality score of 20 (i.e., with a ≤1% probability of being incorrect), which accounted for 90% (Cancer Hotspot Panel)/ 75% (Custom Panel) of the total reads, were selected. Pathological variants in the 50 cancer-related genes were defined using the same criteria as reported previously (1-4), and "high-impact variants" such as frameshift, stop gain, stop loss, and start loss were defined in SnpEff v4.3 (5) for all exon sequences in the Ion AmpliSeq™ Custom Panel, in addition to pathogenic/oncogenic variants in the ClinVar (6) and OncoKB (7) databases.

Immunohistochemistry (IHC) for protein 53 (p53) and mismatch repair (MMR) protein in the NCCH cohort

All surgically resected specimens were fixed in 10% neutral-buffered formalin for 24–72 h and embedded in paraffin. Representative whole 4 μm-thick sections were analyzed using IHC. The following antibodies were used for IHC

on representative slides for each case: anti-p53 (DO7, pre-diluted; Dako, Glostrup, Denmark), anti-hMLH1 (ES05, 1:200 dilution; Dako), anti-hMSH2 and anti-hMSH6 (SP93, 1:200 dilution; Spring Bioscience, CA, USA), and anti-hPMS2 (A16-4, 1:200 dilution; Biocare Medical, CA, USA). All IHC assays were performed using a Dako autostainer (Dako), according to the manufacturer's instructions. After deparaffinization, the tissue sections were stained using the antibodies indicated above and counterstained with hematoxylin. An aberrant p53 staining pattern was defined as a strong and diffuse nuclear staining pattern (> 80% of carcinoma cells) or completely negative ("null pattern") staining of carcinoma cells, with appropriate staining of the surrounding non-tumor cells as an internal positive control. A weak and heterogeneous staining pattern of the tumor cells was classified as wild type (8). Since IHC for PMS2 and MSH6 alone can reportedly replace the four antibody panels (comprising MLH1, MSH2, MSH6, and PMS2) for deficient MMR screening (9,10), MMR-deficient status was defined as the complete loss of nuclear staining for PMS2 and/or MSH6 proteins. Adjacent normal mucosa, stromal cells, and inflammatory cells with intact nuclear staining served as internal positive controls.

Identification of novel stratified genes in the NSMP group in the NCCH cohort

Among the recurrent mutated genes for which subgroup analysis was possible, we first identified single genes significantly associated with prognosis. Then, we investigated combinations that could be better stratified for prognosis than single genes, resulting in the selection of *ARID1A* (36.9%) and *KRAS* (27.2%).

Genome profiling data obtained from the Center for Cancer Genomics and Advanced Therapeutics (C-CAT) database

The genomic profiles of 922 advanced endometrial carcinoma cases were obtained by controlled access to the C-CAT database for this study (11,12). The profiles were obtained with two comprehensive genomic profiling tests approved by the Pharmaceuticals and Medical Devices Agency: FoundationOne CDx, which can detect somatic mutations in 324 genes, and NCC Oncopanel, which detected somatic and germline mutations in 114 genes. The study protocol was approved by the Institutional Review Board of the National Cancer Center Research Institute (approval number 2020-067), followed by the approval of the C-CAT Data Utilization Review Board (approval number CDU2021-001N), and the study was conducted in accordance with the ethical guidelines of the

Declaration of Helsinki. The COSMIC (version 87) and Clinver (20190114) databases were used to annotate each gene aberration. Somatic mutations were counted if they were defined as "pathogenic/likely pathogenic variants" or "oncogenic/likely oncogenic variants".

Molecular classifiers in each cohort

The molecular classification was determined based on IHC for the NCCH cohort and sequencing data provided by the Foundation (FoundationOne CDx or NCC Oncopanel) for the C-CAT cohort. The molecular classification of each cohort is shown in below.

Subtype	Molecular classification (NCCH cohort)	Sequence-based classification (C-CAT cohort)
POLE-EDM	Pathogenic/oncogenic variants detected within the exonuclease domain	
MMR-D/MSI-H	IHC	MSI-H by each panel test
p53abn/*TP53*mut	IHC	Pathogenic/Oncogenic variants
NSMP	The remainder are currently classified as NSMP	

Comparison of genetic alterations between the NCCH and the C-CAT cohorts

In the NCCH cohort, several genes (e.g., *PIK3CA*, *KRAS*, and *ATM*) were targeted only to the hotspot using the Ion AmpliSeq™ cancer hotspot panel v2; thus, comparisons of genetic alterations between the two cohorts for these genes were aligned to the hotspots BED region.

Reference:

1. Hirose S, Murakami N, Takahashi K, Kuno I, Takayanagi D, Asami Y, *et al.* Genomic alterations in STK11 can predict clinical outcomes in cervical cancer patients. *Gynecol Oncol* 2020;**156**(1):203-10.
2. Kuno I, Takayanagi D, Asami Y, Murakami N, Matsuda M, Shimada Y, *et al.* TP53 mutants and non-HPV16/18 genotypes are poor prognostic factors for concurrent chemoradiotherapy in locally advanced cervical cancer. *Sci Rep* 2021;**11**(1):19261.
3. Murakami N, Asami Y, Yoshida H, Takayanagi D, Hirose S, Kuno I, *et al.* Distribution of genetic alterations in high-risk early-stage cervical cancer patients treated with postoperative radiation therapy. *Sci Rep* 2021;**11**(1):10567.
4. Takayanagi D, Hirose S, Kuno I, Asami Y, Murakami N, Matsuda M, *et al.* Comparative Analysis of Genetic Alterations, HPV-Status, and PD-L1 Expression in Neuroendocrine Carcinomas of the Cervix. *Cancers (Basel)* 2021;**13**(6):1215.
5. Cingolani P, Platts A, Wang le L, Coon M, Nguyen T, Wang L, *et al.* A program for annotating and predicting the effects of single nucleotide polymorphisms, SnpEff: SNPs in the genome of Drosophila melanogaster strain w1118; iso-2; iso-3. *Fly (Austin)* 2012;**6**(2):80-92.
6. Landrum MJ, Lee JM, Benson M, Brown GR, Chao C, Chitipiralla S, *et al.* ClinVar: improving access to variant interpretations and supporting evidence. *Nucleic Acids Res* 2018;**46**(D1):D1062-D7.
7. Chakravarty D, Gao J, Phillips SM, Kundra R, Zhang H, Wang J, *et al.* OncoKB: A Precision Oncology Knowledge Base. *JCO Precis Oncol* 2017;**2017**:PO.17.00011.
8. Talhouk A, McConechy MK, Leung S, Li-Chang HH, Kwon JS, Melnyk N, *et al.* A clinically applicable molecular-based classification for endometrial cancers. *Br J Cancer* 2015;**113**(2):299-310.
9. Hall G, Clarkson A, Shi A, Langford E, Leung H, Eckstein RP, *et al.* Immunohistochemistry for PMS2 and MSH6 alone can replace a four antibody panel for mismatch repair deficiency screening in colorectal adenocarcinoma. *Pathology* 2010;**42**(5):409-13.
10. Mojtahed A, Schrijver I, Ford JM, Longacre TA, Pai RK. A two-antibody mismatch repair protein immunohistochemistry screening approach for colorectal carcinomas, skin sebaceous tumors, and gynecologic tract

carcinomas. *Mod Pathol* 2011;**24**(7):1004-14.
11. Mukai Y, Ueno H. Establishment and implementation of Cancer Genomic Medicine in Japan. *Cancer Sci* 2021;**112**(3):970-7.
12. Ebi H, Bando H. Precision Oncology and the Universal Health Coverage System in Japan. *JCO Precis Oncol* 2019;**3**:PO.19.00291.

Supplementary Table S1. Patient characteristics according to molecular subtypes in NCCH cohort.

Characteristics	All		*POLE* -EDM		MMR-D		NSMP		p53abn		*P* value
	[n=265]	(%)	[n=36]	(13.6%)	[n=70]	(26.4%)	[n=103]	(38.9%)	[n=56]	(21.1%)	
Clinicopathological parameters											
Age [year] (median,range)	57	(28-89)	56	(38-80)	54	(28-89)	56	(29-86)	65	(35-84)	<0.001*
Histological types											<0.001**
Endometrioid											
Grade 1	92	(34.7%)	13	(36.0%)	21	(30.0%)	54	(52.4%)	4	(7.1%)	
Grade 2	30	(11.3%)	2	(5.6%)	10	(14.3%)	17	(16.5%)	1	(1.8%)	
Grade 3	76	(28.7%)	17	(47.2%)	31	(44.3%)	17	(16.5%)	11	(19.7%)	
Carcinosarcoma	23	(8.7%)	1	(2.8%)	2	(2.9%)	6	(5.8%)	14	(25.0%)	
Serous	18	(6.8%)	0	(0.0%)	1	(1.4%)	1	(1.0%)	16	(28.6%)	
Clear	12	(4.4%)	1	(2.8%)	0	(0.0%)	7	(6.8%)	4	(7.1%)	
Mixed	11	(4.2%)	2	(5.6%)	4	(5.7%)	0	(0.0%)	5	(8.9%)	
Others	3	(1.2%)	0	(0.0%)	1	(1.4%)	1	(1.0%)	1	(1.8%)	
FIGO (2008) stage											0.065**
IA	99	(37.4%)	20	(55.6%)	26	(37.2%)	34	(33.0%)	19	(33.9%)	
IB	41	(15.5%)	6	(16.7%)	13	(18.6%)	16	(15.5%)	6	(10.7%)	
II	22	(8.3%)	3	(8.3%)	5	(7.1%)	12	(11.6%)	2	(3.6%)	
IIIA	16	(6.0%)	0	(0.0%)	3	(4.3%)	7	(6.8%)	6	(10.7%)	
IIIB	4	(1.5%)	0	(0.0%)	1	(1.4%)	1	(1.0%)	2	(3.6%)	
IIIC	58	(21.9%)	7	(19.4%)	17	(24.3%)	25	(24.3%)	9	(16.1%)	
IVB	25	(9.4%)	0	(0.0%)	5	(7.1%)	8	(7.8%)	12	(21.4%)	
Lymph node metastasis											0.823**
Negative	186	(70.2%)	27	(75.0%)	50	(71.4%)	72	(69.9%)	37	(66.1%)	
Positive	79	(29.8%)	9	(25.0%)	20	(28.6%)	31	(30.1%)	19	(33.9%)	
Myometrial invasion											0.321**
<50%	128	(48.3%)	21	(58.3%)	37	(52.9%)	47	(45.6%)	23	(41.1%)	
≥50%	137	(51.7%)	15	(41.7%)	33	(47.1%)	56	(54.4%)	33	(58.9%)	
Lymph vascular space invasion											0.521**
Negative	111	(41.9%)	16	(44.4%)	24	(34.3%)	46	(44.7%)	25	(44.6%)	
Positive	154	(58.1%)	20	(55.6%)	46	(65.7%)	57	(55.3%)	31	(55.4%)	
Peritoneal cytology											0.047**
Negative	186	(70.2%)	31	(86.1%)	48	(68.6%)	74	(71.8%)	33	(58.9%)	
Positive	79	(29.8%)	5	(13.9%)	22	(31.4%)	29	(28.2%)	23	(41.1%)	
Cervical invasion											0.189**
Negative	202	(76.2%)	32	(88.9%)	55	(78.6%)	75	(72.8%)	40	(71.4%)	
Positive	63	(23.8%)	4	(11.1%)	15	(21.4%)	28	(27.2%)	16	(28.6%)	
Recurrence or progression											<0.001**
None	192	(72.5%)	35	(97.2%)	53	(75.7%)	77	(74.8%)	27	(48.2%)	
Recurrence	67	(25.3%)	1	(2.8%)	16	(22.9%)	25	(24.2%)	25	(44.6%)	
Progression	6	(2.2%)	0	(0.0%)	1	(1.4%)	1	(1.0%)	4	(7.2%)	
Follow-up period [month] (median, range)	61	(3-149)	62	(29-135)	63	(3-138)	61	(5-149)	56	(6-114)	0.063*

* Mann-Whitney's U test. ** Chi-squared test.

NCCH: National Cancer Center Hospital, *POLE* -EDM: DNA polymerase epsilon exonuclease domain mutation, MMR-D: Mismatch repair protein deficiency, NSMP: no specific molecular profile, p53abn: p53 abnormal expression, FIGO: International Federation of Gynecology and Obstetrics.

Supplementary Table S2. Clinicopathological features of 57 cases with discrepancy between p53 IHC status and *TP53* mutations in NCCH cohort.

Characteristics	*TP53* mut / p53wt				*TP53* wt / p53abn				*P* value
	[n=42]	(	%	)	[n=15]	(	%	)	
Molecular subtype									<0.001
POLE-EDM	12	(	28.6%	)	0	(	0.0%	)	
MMR-D	8	(	19.0%	)	7	(	46.7%	)	
NSMP	22	(	52.4%	)	0	(	0.0%	)	
p53abn	0	(	0.0%	)	8	(	53.3%	)	
Histology									0.011
Endometrioid									
Grade 1	11	(	26.2%	)	1	(	6.7%	)	
Grade 2	6	(	14.2%	)	0	(	0.0%	)	
Grade 3	20	(	47.6%	)	5	(	33.3%	)	
Carcinosarcoma	1	(	2.4%	)	3	(	20.0%	)	
Serous	1	(	2.4%	)	2	(	13.3%	)	
Clear	2	(	4.8%	)	3	(	20.0%	)	
Mixed	1	(	2.4%	)	0	(	0.0%	)	
Others	0	(	0.0%	)	1	(	6.7%	)	
FIGO (2008) stage									0.113
I&II	24	(	57.1%	)	5	(	33.3%	)	
III&IV	18	(	42.9%	)	10	(	66.7%	)	
Recurrence or progression									0.143
None	31	(	73.8%	)	8	(	53.3%	)	
Recurrence	10	(	23.8%	)	7	(	46.7%	)	
Progression	1	(	2.4%	)	0	(	0.0%	)	
Death									0.358
Negative	33	(	78.6%	)	10	(	66.7%	)	
Positive	9	(	21.4%	)	5	(	33.3%	)	

NCCH: National Cancer Center Hospital, *TP53* mut: *TP53* mutation, p53wt: p53 wild type, *POLE*-EDM: DNA polymerase epsilon exonuclease domain mutation, MMR-D: Mismatch repair protein deficiency, NSMP: no specific molecular profile, p53abn: p53 abnormal expression, FIGO: International Federation of Gynecology and Obstetrics.

Supplementary Table S3. Hazard ratios for relapse-free survival and overall survival according to prognostic factors in 259 patients with endometrial cancer in NCCH cohort.

Survival	Variable	Event/n	Univariate					Multivariate				
			HR	(	95% CI	)	*P* value	HR	(	95% CI	)	*P* value
Relapse free	Age [year] (≥65/<65)	20/75	1.01	(	0.60-1.71	)	0.965					
	FIGO (2008) stage (III&IV/I&II)	44/97	4.19	(	2.53-6.96	)	<0.001	3.57*	(	2.14-5.97	)	<0.001
	Histological grade (high/low)[1]	44/137	1.88	(	1.13-3.11	)	0.015	1.33*	(	0.74-2.40	)	0.335
	Molecular subtype											
	POLE -EDM	1/36			Reference					Reference		
	MMR-D	16/69	10.0	(	1.33-75.5	)	0.026	8.45*	(	1.12-63.8	)	0.039
	NSMP	25/102	10.3	(	1.39-75.7	)	0.023	8.85*	(	1.19-65.9	)	0.033
	p53abn	25/52	23.6	(	3.19-174.1	)	0.002	16.2*	(	2.16-121.0	)	0.007
	Somatic alterations (mut/wt)											
	PTEN	25/147	0.41	(	0.25-0.67	)	<0.001	0.42**	(	0.25-0.69	)	<0.001
	ARID1A	21/112	0.58	(	0.35-0.97	)	0.038	0.58**	(	0.35-0.97	)	0.040
Overall	Age [year] (≥65/<65)	13/75	1.04	(	0.54-2.00	)	0.898					
	FIGO (2008) stage (III&IV/I&II)	32/97	5.90	(	2.97-11.7	)	<0.001	5.86***	(	2.94-11.7	)	<0.001
	Histological grade (high/low)[1]	31/137	2.40	(	1.23-4.68	)	0.010	1.90***	(	0.97-3.74	)	0.062
	Molecular subtype											
	POLE -EDM	0/36			Reference							
	MMR-D	10/69	1.43E+09	(	-	)	1.00					
	NSMP	17/102	1.74E+09	(	-	)	1.00					
	p53abn	16/52	3.71E+09	(	-	)	1.00					
	Somatic alterations (mut/wt)											
	PTEN	17/147	0.45	(	0.24-0.83	)	0.011	0.47***	(	0.25-0.87	)	0.017
	ARID1A	14/112	0.63	(	0.33-1.19	)	0.153					

*Adjusted by FIGO stage, histological grade, and molecular subtype. **Adjusted by molecular subtype, FIGO stage, histological grade, and *PTEN* or *ARID1A* mutation.
***Adjusted by FIGO stage, histological type, and *PTEN* mutation.

[1] high: endometrioid carcinoma grade3, carcinosarcoma, serous, clear, mixed, and others, low: endometrioid carcinoma grade1 or 2.
NCCH: National Cancer Center Hospital, HR: Hazard ratio, CI: Confidence interval, FIGO: International Federation of Gynecology and Obstetrics, *POLE* -EDM: DNA polymerase epsilon exonuclease domain mutation, MMR-D: Mismatch repair protein deficiency, NSMP: no specific molecular profile, p53abn: p53 abnormal expression, mut: mutant, wt: wild type.

Supplementary Table S4. Hazard ratios for clinical outcomes according to prognostic factors or somatic mutations in patients with NSMP in NCCH cohort.

Survival	Variable	Event/n	Univariate					Multivariate*				
			HR	(	95% CI	)	*P* value	HR	(	95% CI	)	*P* value
Relapse free	Age [year] (≥65/<65)	7/25	1.34	(	0.56-3.24	)	0.514					
	FIGO (2008) stage (III&IV/I&II)	17/40	4.22	(	1.81-9.81	)	<0.001	3.35	(	1.40-8.00	)	0.007
	Histological grade (high/low)[1]	13/31	2.95	(	1.34-6.51	)	0.007	1.78	(	0.78-4.09	)	0.174
	Genes detected in somatic mutation (mut/wt)											
	KRAS + ARID1A											
	KRAS wt + *ARID1A* mut	2/27			Reference					Reference		
	KRAS wt + *ARID1* A wt	11/47	3.32	(	0.73-15.0	)	0.119	2.53	(	0.56-11.5	)	0.231
	KRAS mut + *ARID1A* mut	2/11	2.81	(	0.39-20.0	)	0.303	1.52	(	0.21-11.2	)	0.681
	KRAS mut + *ARID1A wt*	10/17	11.0	(	2.39-50.3	)	0.002	6.98	(	1.47-33.2	)	0.015
Overall	Age [year] (≥65/<65)	5/25	1.53	(	0.53-4.41	)	0.435					
	FIGO (2008) stage (III&IV/I&II)	14/40	8.28	(	2.37-28.9	)	<0.001	5.54	(	1.54-20.0	)	0.009
	Histological grade (high/low)[1]	12/31	5.89	(	2.07-16.8	)	<0.001	3.49	(	1.18-10.3	)	0.024
	Genes detected in somatic mutation (mut/wt)											
	KRAS + ARID1A											
	KRAS wt + *ARID1A* mut	1/27			Reference					Reference		
	KRAS wt + *ARID1* A wt	6/47	3.21	(	0.38-26.9	)	0.283	1.99	(	0.24-16.9	)	0.526
	KRAS mut + *ARID1A* mut	2/11	5.41	(	0.49-60.0	)	0.169	1.75	(	0.15-20.8	)	0.659
	KRAS mut + *ARID1A wt*	8/17	14.9	(	1.86-120.0	)	0.011	5.99	(	0.71-50.7	)	0.100

*Adjusted by age, FIGO stage, and histological type.

[1] high: endometrioid carcinoma grade3, carcinosarcoma, serous, clear, mixed, and others, low: endometrioid carcinoma grade1 or 2.

NSMP: no specific molecular profile, NCCH: National Cancer Center Hospital, HR: Hazard ratio, CI: Confidence interval, FIGO: International Federation of Gynecology and Obstetrics, mut: mutant, wt: wild type.

Supplementary Table S5.Clinicopathological characteristics of 17 cases with *KRAS* mutated and *ARID1A* wild type in NCCH cohort.

Patients	Age	Histology	FIGO	Recurrence status	Metastatic lesions	Outcome	Adjuvant therapy
1	46	EEC G2	IVB	Recurrence	Distant (lung)	DOD	TAP
2	49	EEC G3	IIIA	Recurrence	Distant (bone)	DOD	AP
3	37	CS	IIIC1	Recurrence	Distant (lung)	DOD	IFM+PTX
4	46	CS	IIIC2	Recurrence	Distant (cancerous pleurisy)	DOD	IFM+PTX
5	48	CS	IA	Recurrence	Distant (lung)	DOD	IFM+PTX
6	63	Serous	IIIC1	Recurrence	Distant (PALN→brain)	DOD	AP
7	61	EEC G2	IIIC1	Recurrence	Local (vagina)	DOD	DP
8	62	EEC G2	IIIC1	Recurrence	Local (vagina)	DOC	AP
9	49	EEC G1	II	Recurrence	Distant (lung)	AWD	AP
10	77	EEC G1	IB	Recurrence	Local (vagina)	AWD	None
11	65	EEC G1	IB	No	-	NED	None
12	53	EEC G1	IA	No	-	NED	None
13	53	EEC G1	IA	No	-	NED	None
14	47	EEC G1	IA	No	-	NED	None
15	60	EEC G3	IB	No	-	NED	None
16	47	EEC G3	IIIC1	No	-	NED	AP
17	51	Others	IA	No	-	NED	None

NCCH: National Cancer Center Hospital, FIGO: International Federation of Gynecology and Obstetrics, EEC G: endometrioid endometrial carcinoma grade, CS: carcinosarcoma, PALN: para-aortic lymph nodes, DOD: died of disease, AWD: alive with disease, NED: no evidence of disease, TAP: paclitaxel, adriamycin, and cisplatin, AP: adriamycin and cisplatin, IFM+PTX: ifosfamide and paclitaxel, DP: docetaxel and cisplatin.

Supplementary Table S6. Patient characteristics according to molecular subtypes in C-CAT cohort.

Characteristics	All		*POLE*-EDM		MSI-H		NSMP		*TP53* mutation		*P* value
	[n=764]	(%)	[n=16]	(2.1%)	[n=87]	(11.4%)	[n=267]	(34.9%)	[n=394]	(51.6%)	
Clinicopathological parameters											
Age (median,range)	63	(25-85)	58	(38-78)	55	(35-78)	61	(25-82)	65	(30-85)	<0.001*
Histological types											
Endometrioid											
Unknown grade	255	(33.4%)	3	(18.75%)	45	(51.7%)	128	(48.0%)	79	(20.0%)	
Grade 1	27	(3.5%)	0	(0.0%)	2	(2.3%)	19	(7.1%)	6	(1.5%)	
Grade 2	31	(4.1%)	1	(6.25%)	1	(1.2%)	18	(6.7%)	11	(2.8%)	
Grade 3	43	(5.6%)	4	(25.0%)	7	(8.0%)	16	(6.0%)	16	(4.0%)	
Serous	132	(17.3%)	1	(6.25%)	2	(2.3%)	8	(3.0%)	121	(30.7%)	
Carcinosarcoma	130	(17.0%)	1	(6.25%)	6	(6.9%)	29	(10.9%)	94	(23.9%)	
Mixed	26	(3.4%)	0	(0.0%)	6	(6.9%)	3	(1.1%)	17	(4.3%)	
Clear	25	(3.3%)	1	(6.25%)	0	(0.0%)	13	(4.9%)	11	(2.8%)	
Un/De-differentiated	18	(2.4%)	1	(6.25%)	6	(6.9%)	8	(3.0%)	3	(0.8%)	
Neuroendocrine	7	(0.9%)	0	(0.0%)	1	(1.2%)	2	(0.7%)	4	(1.0%)	
Poorly differentiated carcinoma[1]	14	(1.8%)	1	(6.25%)	3	(3.4%)	1	(0.4%)	9	(2.3%)	
Carcinoma	46	(6.0%)	2	(12.5%)	6	(6.9%)	18	(6.7%)	20	(5.1%)	
Others carcinoma	10	(1.3%)	1	(6.25%)	2	(2.3%)	4	(1.5%)	3	(0.8%)	
Histological grade											<0.001**
Low	58	(7.6%)	1	(6.3%)	3	(3.5%)	37	(13.8%)	17	(4.3%)	
High	405	(53.0%)	10	(62.5%)	33	(37.9%)	84	(31.5%)	278	(70.6%)	
Unclassifiable	301	(39.4%)	5	(31.2%)	51	(58.6%)	146	(54.7%)	99	(25.1%)	
Outcome											0.598***
Alive	630	(82.5%)	13	(81.2%)	71	(81.6%)	227	(85.0%)	319	(81.0%)	
Death	134	(17.5%)	3	(18.8%)	16	(18.4%)	40	(15.0%)	75	(19.0%)	
Follow-up period [month] (median, range)	27	(1-270)	19	(9-94)	23	(1-99)	33	(1-270)	25	(2-198)	0.017*

* Mann-Whitney's U test. ** Low grade vs high grade, Chi-squared test. *** Chi-squared test.

[1] Poorly differentiated adenocarcinoma cases that did not clearly meet the 2020 WHO classification criteria were described as "poorly differentiated carcinoma".

C-CAT: the Center for Cancer Genomics and Advanced Therapeutics, *POLE*-EDM: DNA polymerase epsilon exonuclease domain mutation, MSI-H: Microsatellite instability high, NSMP: no specific molecular profile.

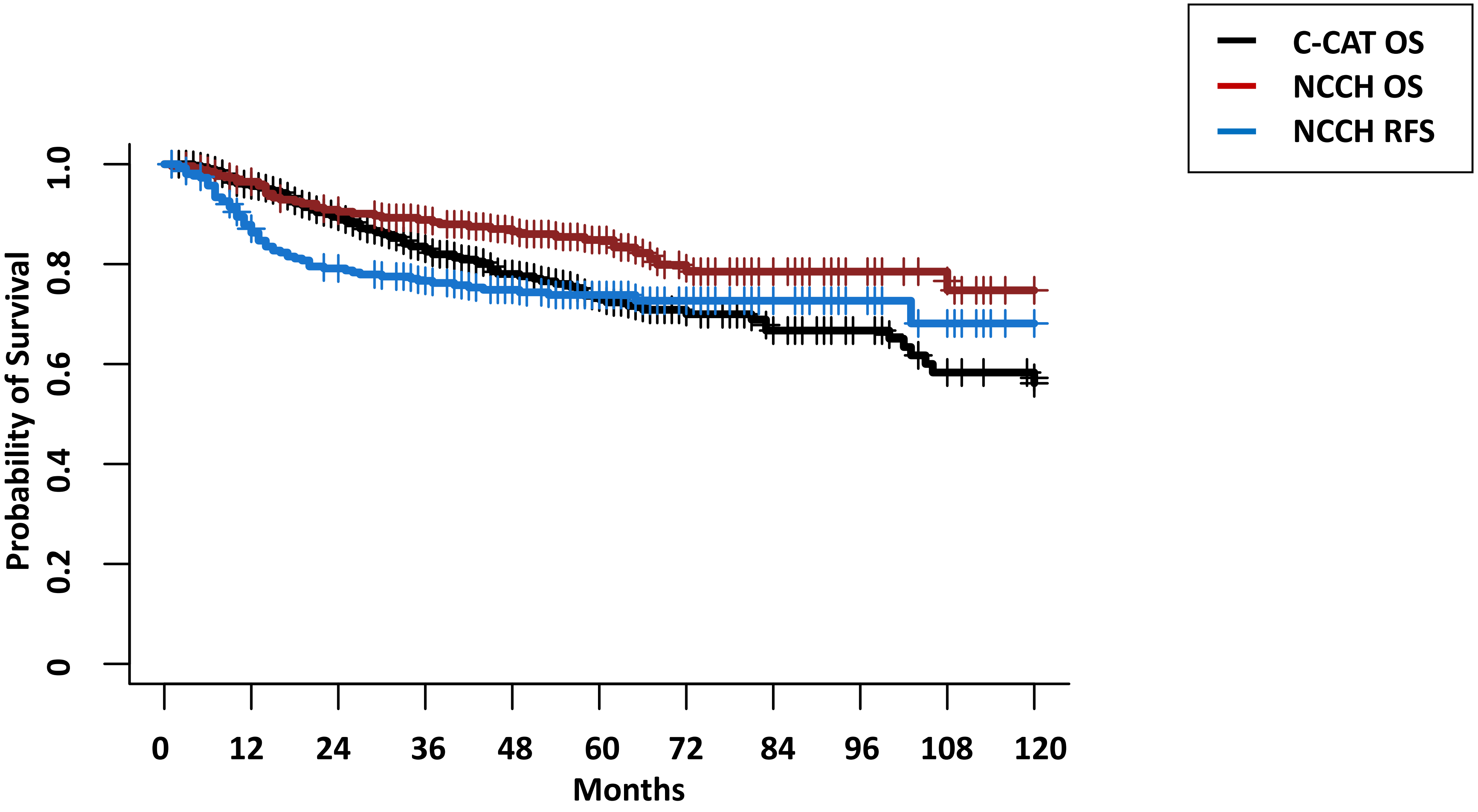

C-CAT OS	718	391	170	78	43	25
NCCH OS	259	224	169	55	27	10
NCCH RFS	259	197	149	46	20	5

Number at risk

Supplementary Figure S1. Kaplan–Meier survival curve for entire cohort.

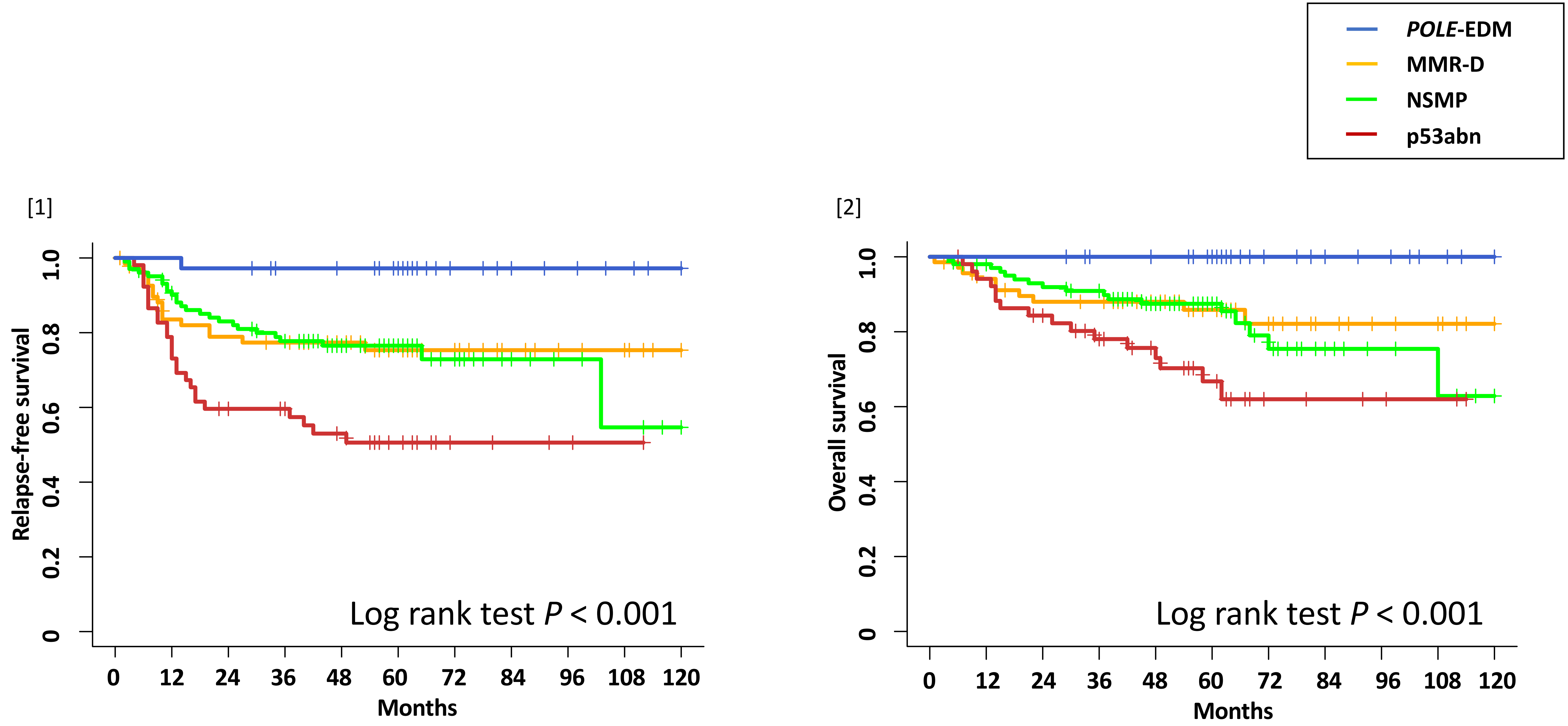

POLE-EDM	36	35	29	10	6	1
MMR-D	69	51	42	19	8	3
NSMP	102	82	58	17	5	1
p53abn	52	30	22	4	2	0

Number at risk

POLE-EDM	36	36	30	10	6	1
MMR-D	69	57	48	22	11	6
NSMP	102	91	66	22	7	3
p53abn	52	42	28	5	3	0

Number at risk

Supplementary Figure S2. Kaplan–Meier survival curves according to molecular subtype at all stages in the NCCH cohort.

[1] RFS and [2] OS.

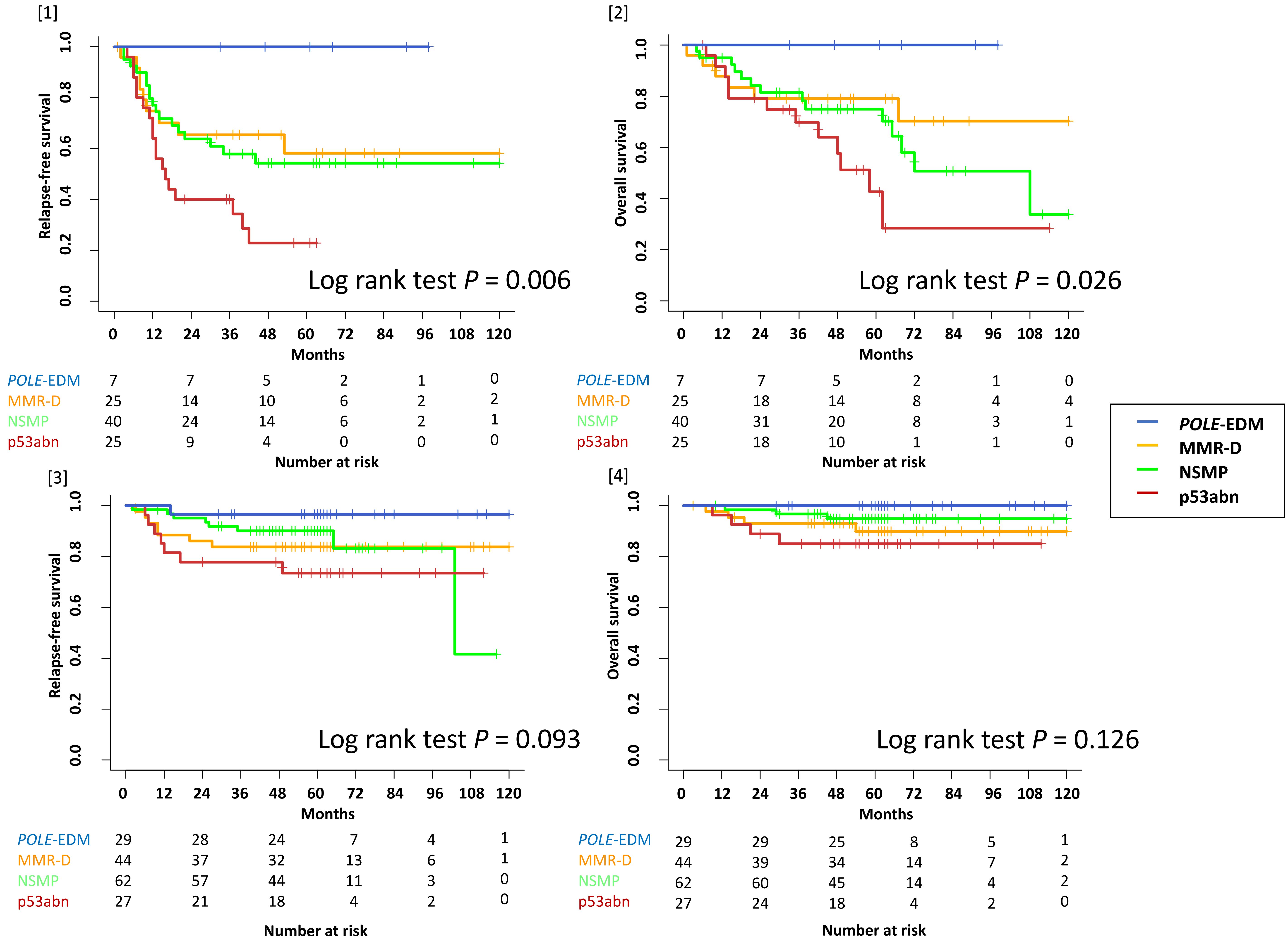

Supplementary Figure S3. Kaplan–Meier survival curves according to molecular subtype by FIGO stages in the NCCH cohort.

[1] RFS in advanced stages (FIGO III–IV). [2] OS in advanced stages. [3] RFS in early stages (FIGO I–II). [4] OS in early stages.

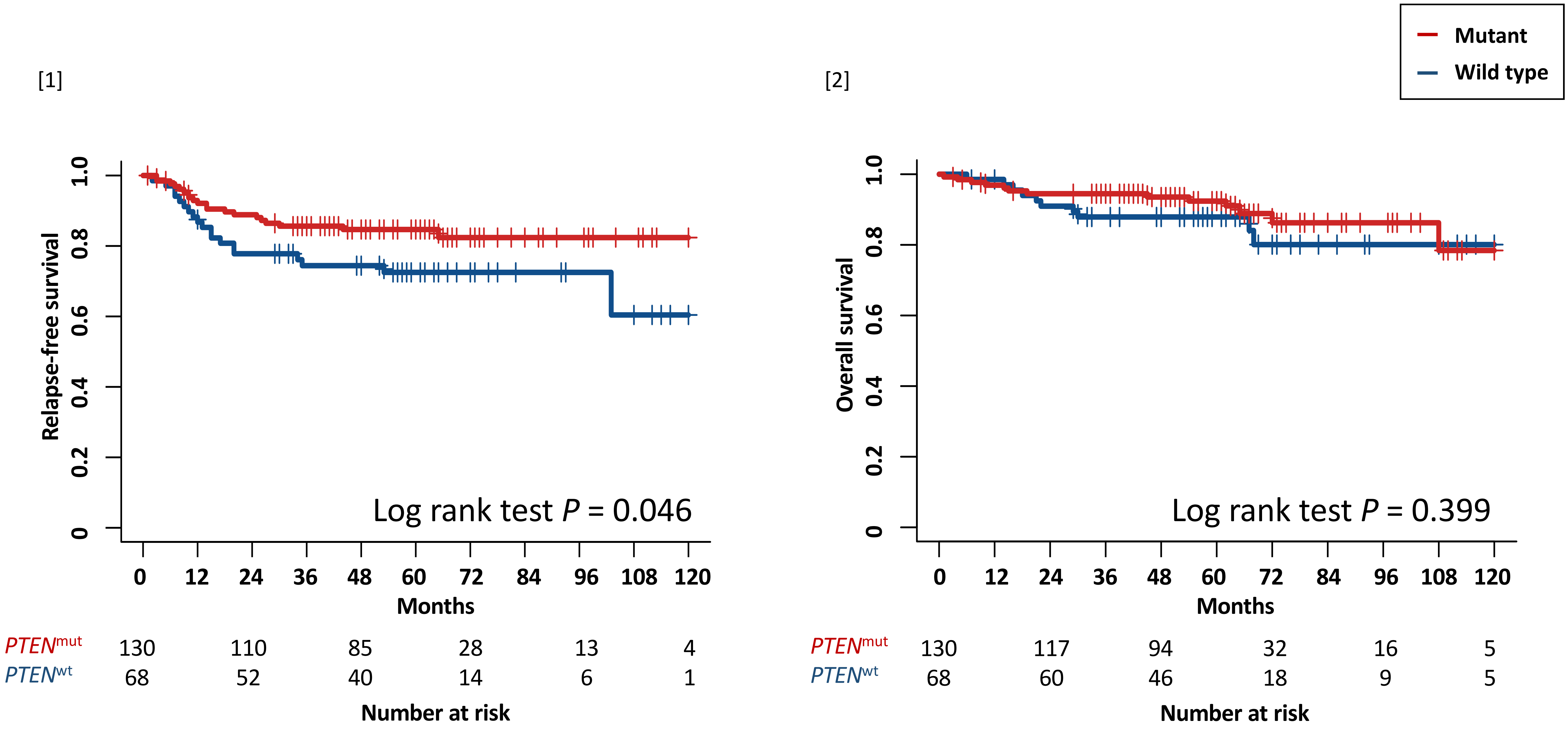

Supplementary Figure S4. Kaplan–Meier survival curves according to *PTEN* mutation status in endometrioid endometrial carcinoma patients in the NCCH cohort.

[1] RFS and [2] OS.

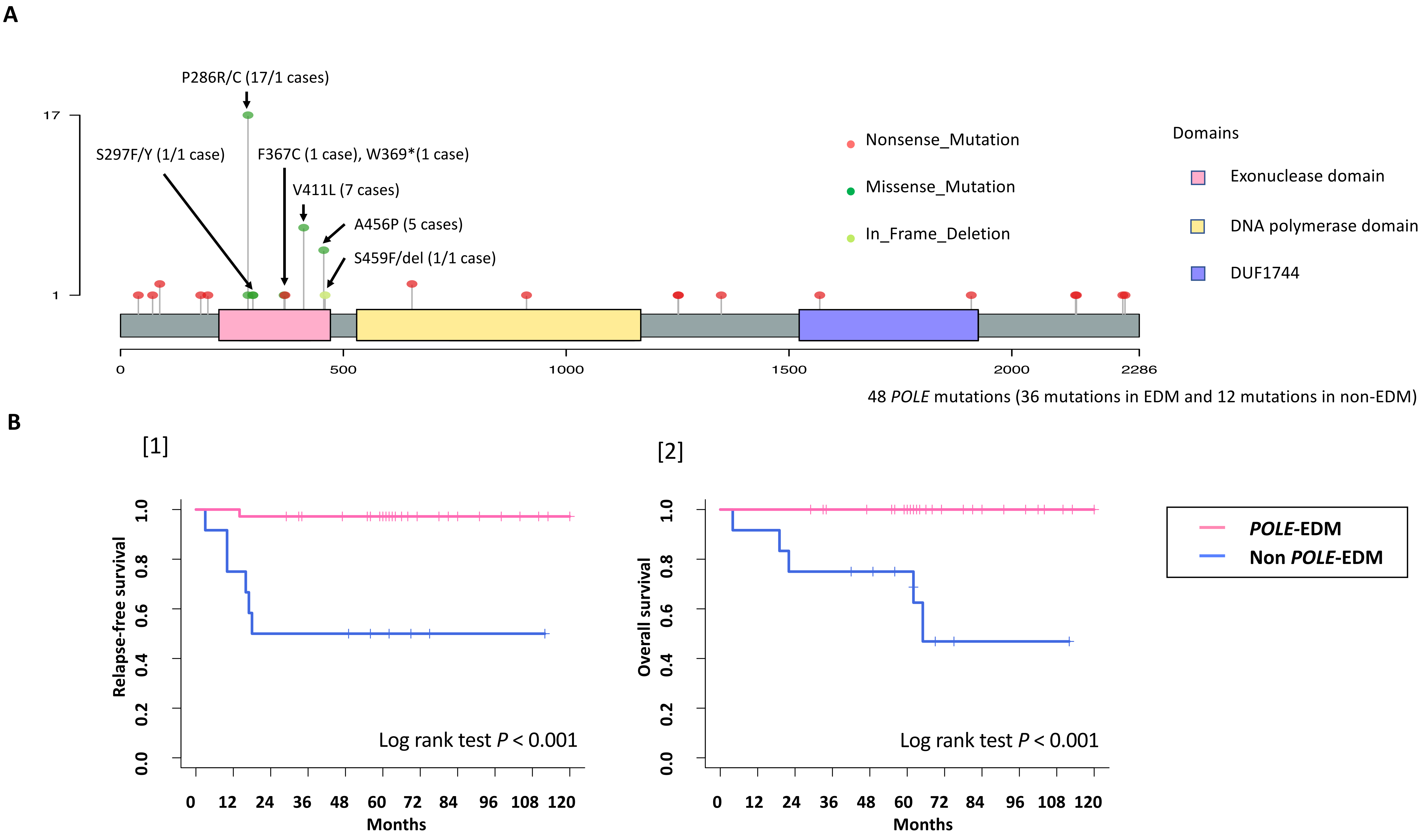

Supplementary Figure S5. *POLE* variants and patient outcomes in the NCCH cohort.

A. Lollipop plot illustrating all *POLE* variants. **B**. Kaplan–Meier survival curves according to *POLE* mutation status. Indicates [1] RFS and [2] OS.

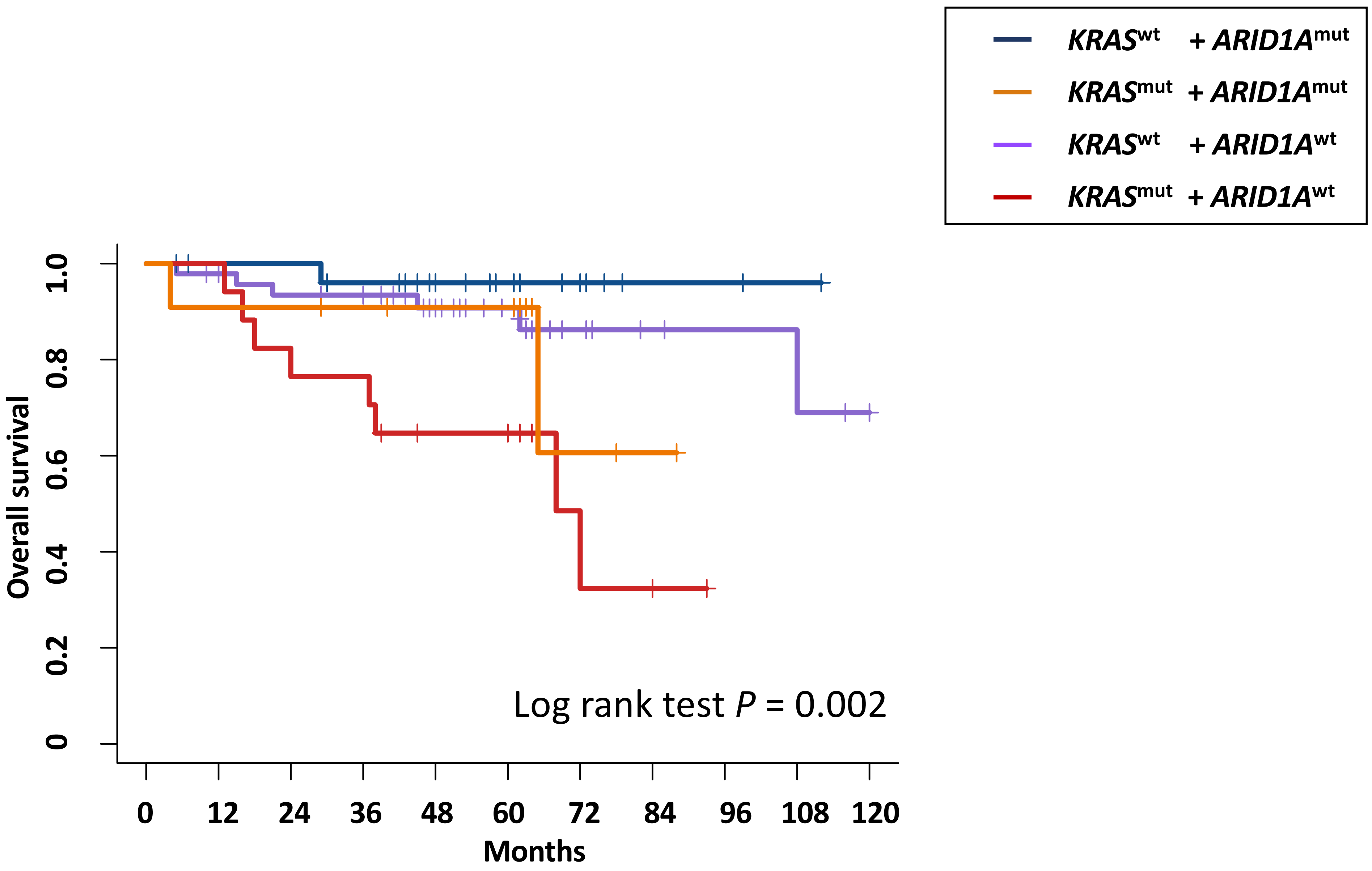

*KRAS*wt + *ARID1A*mut	27	25	18	7	2	0
*KRAS*mut + *ARID1A*mut	11	10	8	2	0	0
*KRAS*wt + *ARID1A*wt	47	42	32	10	5	3
*KRAS*mut + *ARID1A*wt	17	14	8	3	0	0

Supplementary Figure S6. **Kaplan–Meier survival curve according to *KRAS* and *ARID1A* status in endometrial cancer patients with no specific molecular profile in the NCCH cohort.**

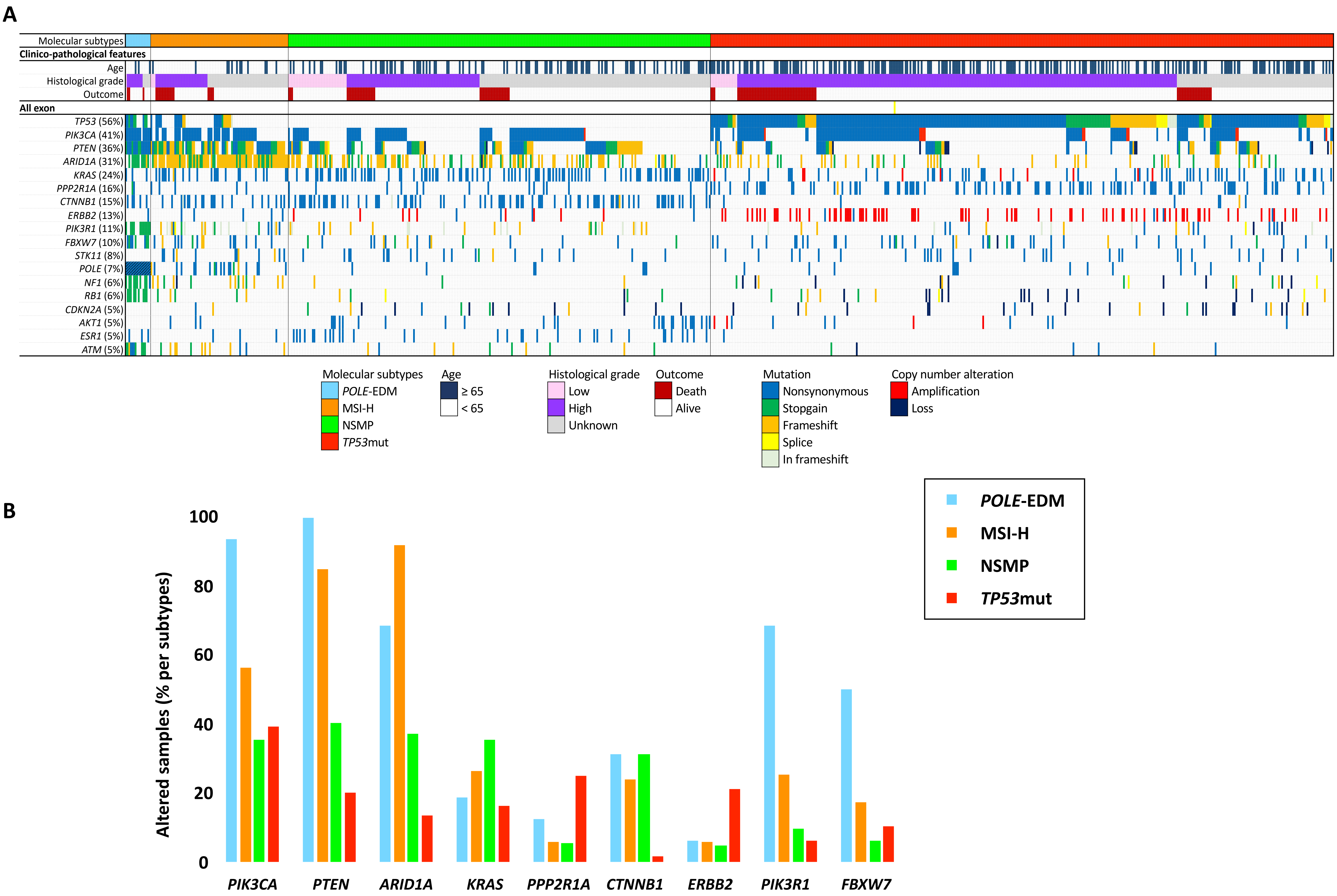

Supplementary Figure S7. Genetic alteration spectrum by molecular subtype in the C-CAT cohort.

A. Clinicopathological factors and molecular subtype. **B**. Differences among the four subgroups of recurrently mutant genes. Shown are the mutation frequencies of all genes that were significantly mutated in at least one of the four subgroups.

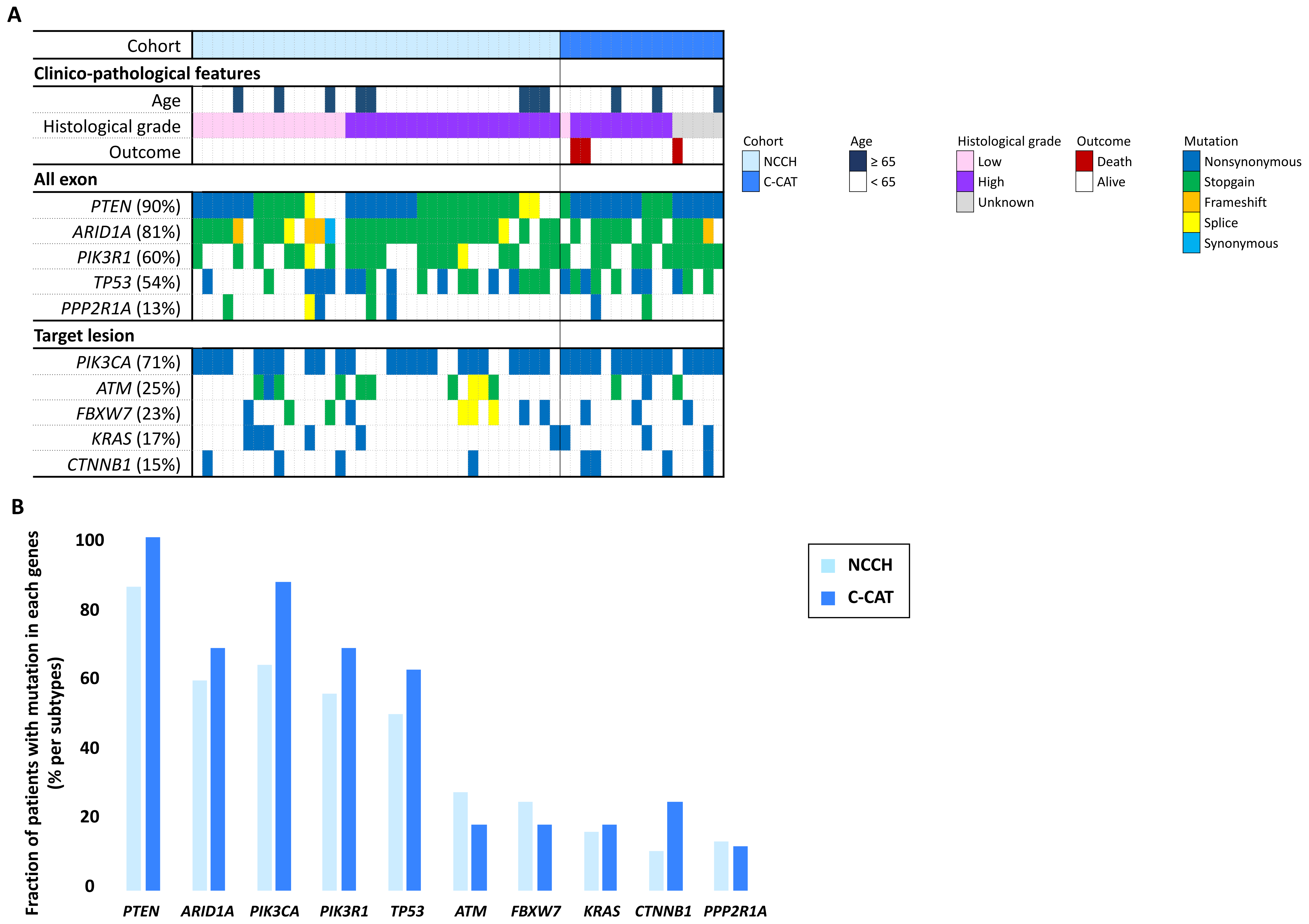

Supplementary Figure S8. Differences in genetic alterations in endometrial cancer patients classified as *POLE*-EDM in the NCCH and C-CAT cohorts.

A. Mutation spectrum. **B**. Differences between the two cohorts of recurrent mutant genes in endometrial cancers with *POLE*-EDM.

A

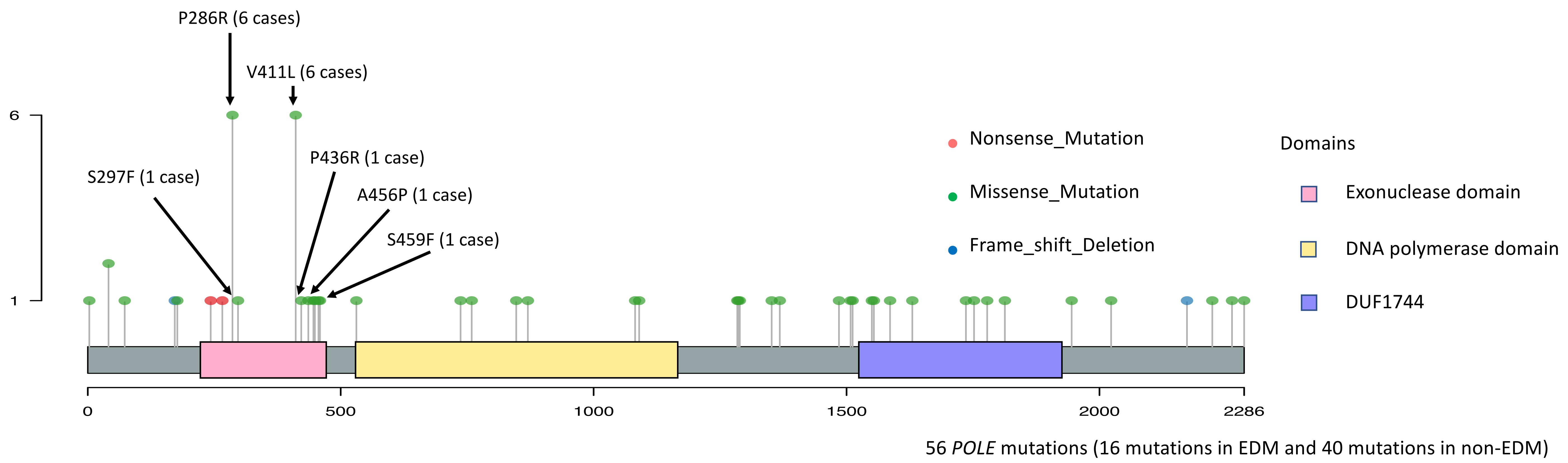

B

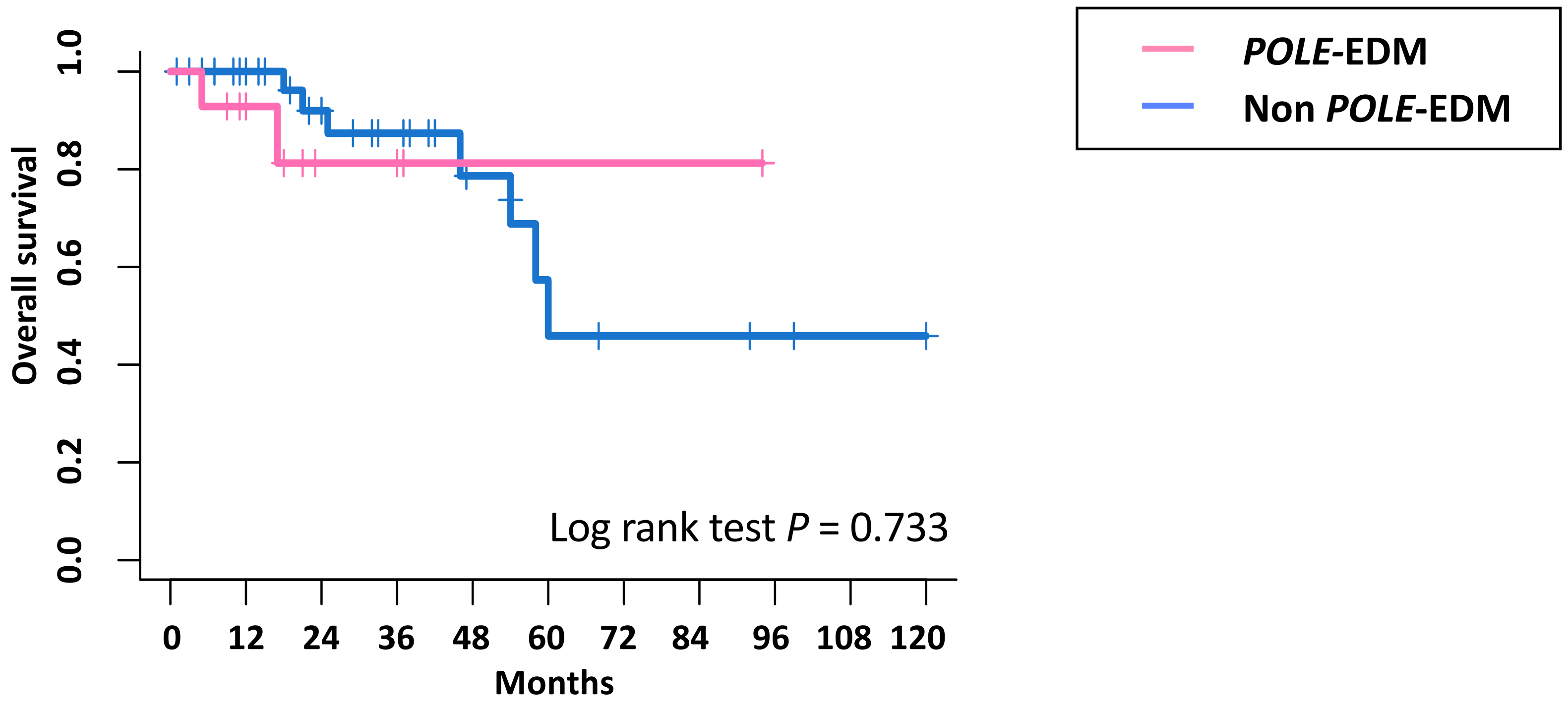

Supplementary Figure S9. *POLE* variants and patient outcome in the C-CAT cohort.

A. Lollipop plot illustrating all *POLE* variants. **B**. Kaplan–Meier survival curve according to *POLE* mutation status.

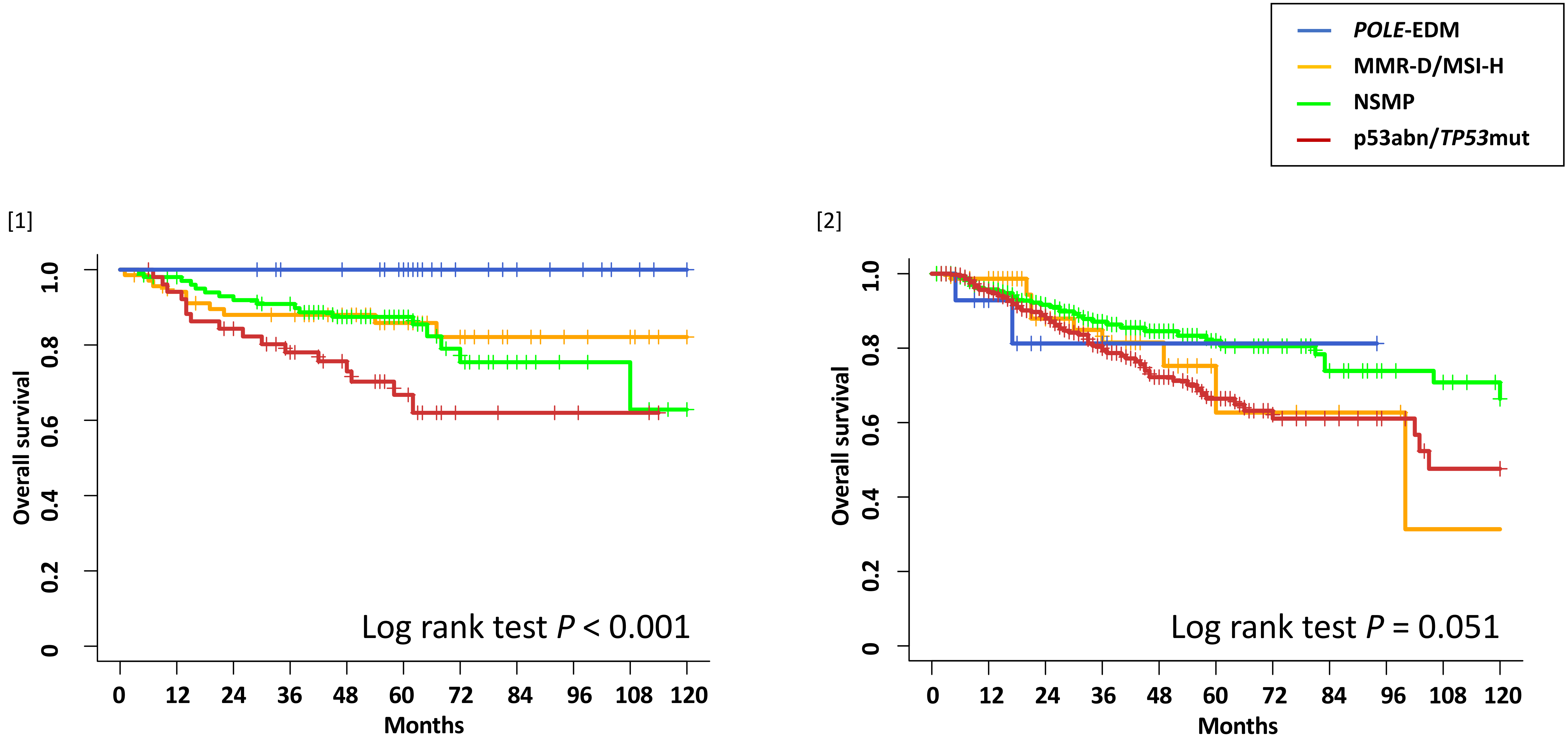

[1]

	0	24	48	72	96	120
POLE-EDM	36	36	30	10	6	1
MMR-D	69	57	48	22	11	6
NSMP	102	91	66	22	7	3
p53abn	52	42	28	5	3	0

Number at risk

[2]

	0	24	48	72	96	120
POLE-EDM	14	4	1	0	0	0
MSI-H	76	36	13	5	3	1
NSMP	252	156	81	44	25	15
*TP53*mut	376	202	76	29	15	9

Number at risk

Supplementary Figure S10. Kaplan-Meier survival curves for overall survival according to molecular subtype in both cohorts.
[1] NCCH cohort and [2] C-CAT cohort.

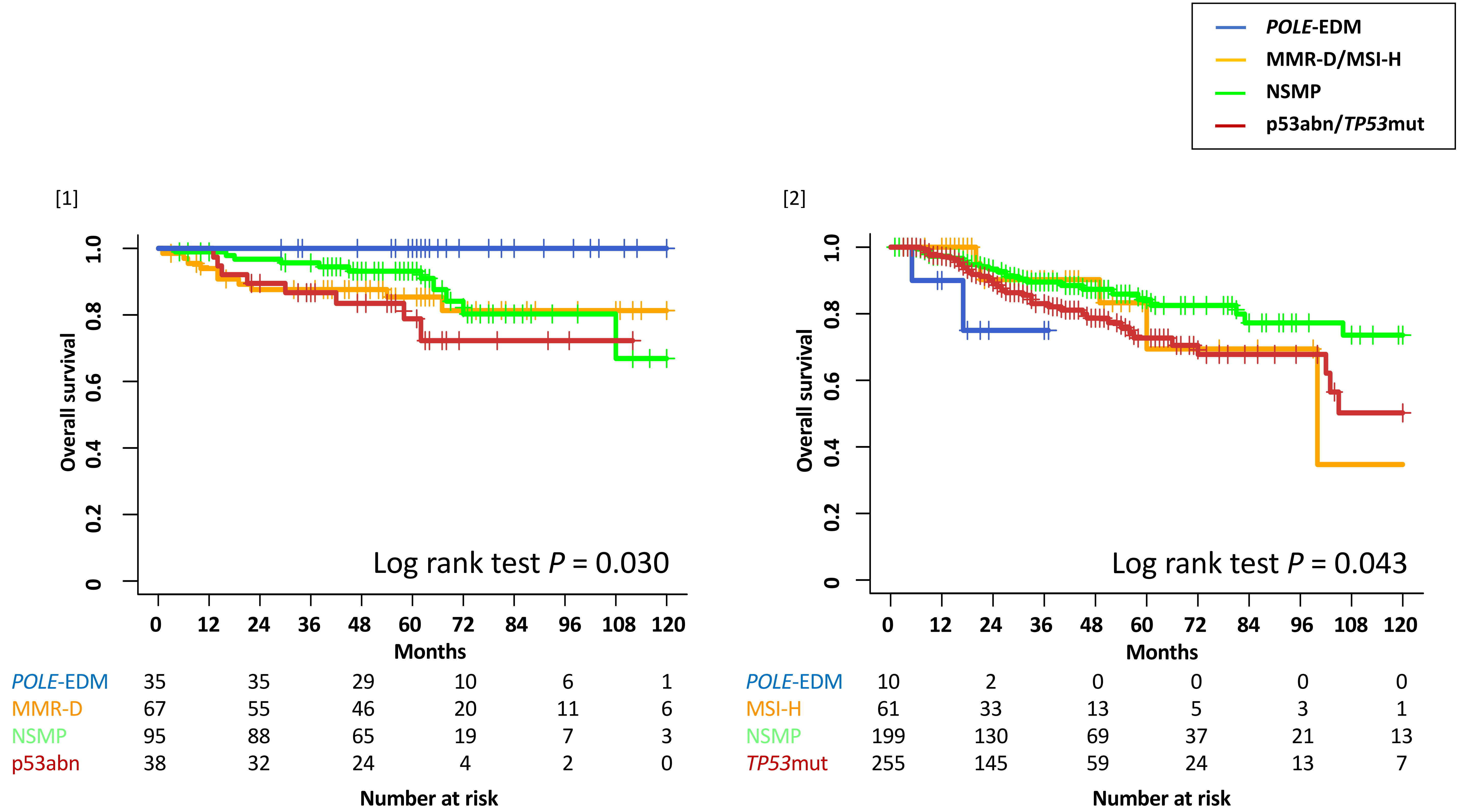

Supplementary Figure S11. Kaplan-Meier survival curves for overall survival according to molecular subtype excluding carcinosarcoma, un/de-differentiated, and carcinomas with unknown histologic details in both cohorts.

[1] NCCH cohort and [2] C-CAT cohort.

Utility of molecular subtypes and genetic alterations for evaluating clinical outcomes in 1,029 patients with endometrial cancer

Asami Y et al.

Supplementary Figure legends

Supplementary Figure S1. Kaplan–Meier survival curve for entire cohort. OS of the C-CAT cohort (black line) and NCCH cohort (red line). RFS of the NCCH cohort (blue line).

C-CAT, the Center for Cancer Genomics and Advanced Therapeutics; NCCH, National Cancer Center Hospital; OS, overall survival; RFS, relapse-free survival.

Supplementary Figure S2. Kaplan–Meier survival curves according to molecular subtype at all stages in the NCCH cohort. [1] RFS of the *POLE*-EDM (blue line), MMR-D (orange line), NSMP (green line), and p53abn (red line) groups. [2] OS of the *POLE*-EDM (blue line), MMR-D (orange line), NSMP (green line), and p53abn (red line) groups.

NCCH, National Cancer Center Hospital; RFS, relapse-free survival; OS, overall survival; *POLE*-EDM, DNA polymerase epsilon exonuclease domain mutation; MMR-D, mismatch repair protein deficiency; NSMP, no specific molecular profile; p53abn, protein 53 abnormal expression.

Supplementary Figure S3. Kaplan–Meier survival curves according to molecular subtype by FIGO stages in the NCCH cohort. [1] RFS in advanced stages (III–IV) of the *POLE*-EDM (blue line), MMR-D (orange line), NSMP (green line), and p53abn (red line) groups. [2] OS in advanced stages of the *POLE*-EDM (blue line), MMR-D (orange line), NSMP (green line), and p53abn (red line) groups. [3] RFS in the early stages (I–II) of the *POLE*-EDM (blue line), MMR-D (orange line), NSMP (green line), and p53abn (red line) groups. [4] OS in the early stages of the *POLE*-EDM (blue line), MMR-D (orange line), NSMP (green line), and p53abn (red line) groups.

NCCH, National Cancer Center Hospital; RFS, relapse-free survival; OS, overall survival; *POLE*-EDM, DNA polymerase epsilon exonuclease domain mutation; MMR-D, mismatch repair deficiency; NSMP, no specific molecular profile; p53abn, protein 53 abnormal expression.

Supplementary Figure S4. Kaplan–Meier survival curves according to *PTEN* mutation status in endometrioid endometrial carcinoma patients in the NCCH cohort. [1] RFS of the *PTEN* mutation (red line) and *PTEN* wild type (blue line) groups. [2] OS of the *PTEN* mutation (red line) and *PTEN* wild type (blue line) groups.
NCCH, National Cancer Center Hospital; RFS, relapse-free survival; OS, overall survival; mut, Mutant; wt, Wild type.

Supplementary Figure S5. *POLE* variants and patient outcomes in the NCCH cohort. **A**. Lollipop plot illustrating all *POLE* variants. **B**. Kaplan–Meier survival curves according to *POLE* mutation status. [1] RFS of the *POLE*-EDM (pink line) and *POLE*-non EDM (dark blue line) groups. [2] OS of the *POLE*-EDM (pink line) and *POLE*-non EDM (dark blue line) groups.
NCCH, National Cancer Center Hospital; RFS, relapse-free survival; OS, overall survival; *POLE*-EDM, DNA polymerase epsilon exonuclease domain mutation.

Supplementary Figure S6. Kaplan–Meier survival curve according to *KRAS* and *ARID1A* status in endometrial cancer patients with no specific molecular profile in the NCCH cohort. OS of the *KRAS* wt and *ARID1A* mutant (navy blue line), *KRAS* mutant and *ARID1A* mutant (dark orange line), *KRAS* wt and *ARID1A* wt (purple line), and *KRAS* mutant and *ARID1A* wt (dark red line) groups.
NCCH, National Cancer Center Hospital; OS, overall survival; mut, Mutant; wt, Wild type.

Supplementary Figure S7. Genetic alteration spectrum by molecular subtype in the C-CAT cohort.
A. Clinicopathological factors and molecular subtypes in the C-CAT cohort. The 764 patients were classified according to histological type, clinicopathological features, and somatic mutations. **B**. Differences among the four subgroups of recurrent mutant genes. Mutation frequencies of all genes that were significantly mutated in at least one of the four subgroups are shown.
C-CAT, the Center for Cancer Genomics and Advanced Therapeutics; *POLE*-EDM, DNA polymerase epsilon exonuclease domain mutation; MSI-H, microsatellite instability high; NSMP, no specific molecular profile; *TP53*mut, *TP53* mutation.

Supplementary Figure S8. Differences in genetic alterations in endometrial cancer patients classified as *POLE*-EDM in the NCCH and C-CAT cohorts. **A**. Mutation spectrum of endometrial cancer patients with *POLE*-EDM. The 52 patients were classified according to histological type, clinicopathological features, and somatic mutations. **B**. Differences between the two cohorts of recurrent mutant genes in endometrial cancer patients with *POLE*-EDM.
POLE-EDM: DNA polymerase epsilon exonuclease domain mutation; NCCH, National Cancer Center Hospital; C-CAT, the Center for Cancer Genomics and Advanced Therapeutics.

Supplementary Figure S9. *POLE* variants and patient outcomes in the C-CAT cohort. **A**. Lollipop plot illustrating all *POLE* variants. **B**. Kaplan–Meier survival curve according to *POLE* mutation status. OS of the *POLE*-EDM (pink line) and *POLE*-non EDM (dark blue line) groups.
C-CAT, the Center for Cancer Genomics and Advanced Therapeutics; OS, overall survival; *POLE*-EDM, DNA polymerase epsilon exonuclease domain mutation.

Supplementary Figure S10. Kaplan–Meier survival curves for overall survival according to molecular subtype in both cohorts. [1] OS of the *POLE*-EDM (blue line), MMR-D (orange line), NSMP (green line), and p53abn (red line) groups in the NCCH cohort. [2] OS of the *POLE*-EDM (blue line), MSI-H (orange line), NSMP (green line), and *TP53*mut (red line) groups in the C-CAT cohort.
NCCH, National Cancer Center Hospital; C-CAT, the Center for Cancer Genomics and Advanced Therapeutics; OS, overall survival; *POLE*-EDM, DNA polymerase epsilon exonuclease domain mutation; MMR-D, mismatch repair deficiency; NSMP, no specific molecular profile; p53abn, protein 53 abnormal expression; MSI-H, microsatellite instability high; *TP53*mut, *TP53* mutation.

Supplementary Figure S11. Kaplan–Meier survival curves for overall survival according to molecular subtype excluding carcinosarcoma, un/de-differentiated, and carcinomas with unknown histologic details in both cohorts. [1] OS of the *POLE*-EDM (blue line), MMR-D (orange line), NSMP (green line), and p53abn (red line) groups in the NCCH cohort. [2] OS of the *POLE*-EDM (blue line), MSI-H (orange line), NSMP (green line), and *TP53*mut (red line) groups in the C-CAT cohort.

NCCH, National Cancer Center Hospital; C-CAT, the Center for Cancer Genomics and Advanced Therapeutics; OS, overall survival; *POLE*-EDM, DNA polymerase epsilon exonuclease domain mutation; MMR-D, mismatch repair deficiency; NSMP, no specific molecular profile; p53abn, protein 53 abnormal expression; MSI-H, microsatellite instability high; *TP53*mut, *TP53* mutation.
